# Superiority of* Chrysophyllum oliviforme* in the green synthesis of highly stable ZnO nanoparticles: metabolomic profiling, quadruple antiviral screening, and comparative MD simulations

**DOI:** 10.1186/s11671-026-04459-z

**Published:** 2026-03-09

**Authors:** Mina Michael Melk, Ahmed F. El-Sayed

**Affiliations:** 1 Pharmacognosy Department, Faculty of Pharmacy, Ahram Canadian University, Giza, Egypt; 2 Microbial Genetics Department, Biotechnology Research Institute, National Research Centre, Giza, Egypt; 3 Egypt Center for Research and Regenerative Medicine (ECRRM), Cairo, Egypt

**Keywords:** Phytochemical profiling, Antiviral, *Chrysophyllum*, Docking, Molecular dynamics

## Abstract

This study presents a comprehensive comparative evaluation of the green synthesis of zinc oxide nanoparticles (ZnO NPs) using alcoholic leaf extracts from *Chrysophyllum oliviforme* and *Chrysophyllum cainito*, focusing on their phytochemical-mediated stabilization and broad-spectrum antiviral efficacy. Ultra-performance liquid chromatography coupled with electrospray ionization time-of-flight mass spectrometry (LC-ESI-TOF-MS) was employed for metabolomic fingerprinting, identifying a total of 86 bioactive metabolites. The profiling revealed that *C. oliviforme* possesses a significantly more robust chemical matrix, characterized by higher relative concentrations of potent antioxidant and antiviral markers, specifically chlorogenic acid (11.43%), quercetin (7.62%), and resveratrol (3.81%), compared to *C. cainito*. The biogenic ZnO NPs were synthesized via a sustainable bottom-up approach, where the identified polyphenolic constituents acted as both reducing and capping agents. Characterization using TEM and SEM imaging, XRD confirmed the formation of crystalline hexagonal structures. Notably, *C. oliviforme*-derived NPs demonstrated superior colloidal stability, evidenced by moderate absolute zeta potential, which correlates with the dense phenolic capping observed in the FTIR spectra. Biological screening against a panel of four diverse viruses, Herpes Simplex Virus type 2 (HSV-2), Human Immunodeficiency Virus type 1 (HIV-1), Avian Influenza (H5N1), and Human Adenovirus-40 (HAdV-40) revealed potent inhibitory activities. The *C. oliviforme* ZnO NPs exhibited the highest therapeutic potential, yielding selectivity indices (SI) of 5.17, 4.44, 8.02, and 4.09, respectively. The MD trajectories (RMSD, RMSF, and Rg) confirmed that these complexes remain stable and compact under physiological conditions. These findings underscore the superiority of *C. oliviforme* as a bio-template for synthesizing highly stable, multi-target antiviral nano-therapeutics.

**Supplementary Information:**

The online version contains supplementary material available at 10.1186/s11671-026-04459-z.

## Introduction

*Chrysophyllum oliviforme* (Satinleaf) and *Chrysophyllum cainito* (star apple) are tropical *Sapotaceae* species valued in traditional medicine and increasingly investigated for their bioactive phytochemicals. Both species are rich sources of secondary metabolites, including flavonoids, phenolic acids, and triterpenoids, which are responsible for a range of pharmacological activities such as antioxidants, anti-inflammatory, and antimicrobial effects [1–4].

Recent advances in green nanotechnology have demonstrated the potential of plant extracts for the biosynthesis of metal nanoparticles, offering a sustainable and non-toxic alternative to conventional chemical methods [5–7]. Extracts from *Chrysophyllum* species have been successfully used to synthesize silver, palladium, and manganese dioxide nanoparticles, highlighting their role as effective reducing and stabilizing agents [8, 9]. However, the phytochemically-mediated synthesis of zinc oxide nanoparticles (ZnO NPs) from these species remains unexplored. ZnO NPs are of significant interest due to their unique optical, catalytic, and biomedical properties, including notable antiviral activity. Zn²⁺ was specifically selected because of its strong coordination affinity with the diverse phenolic and flavonoid hydroxyl groups present in *Chrysophyllum* extracts, which enhances both the reduction and capping efficiency relative to other metals. From a mechanistic perspective, ZnO nanoparticles (NPs) offer a dual advantage: they possess intrinsic antimicrobial activity and serve as stable, biocompatible carriers for the plant’s bioactive antiviral compounds, generating a synergistic effect that can surpass that of silver or palladium nanoparticles within these leaf matrices [9, 10].

Molecular docking and dynamics simulations are indispensable tools in rational drug design, enabling the prediction of interactions between bioactive compounds and target proteins [11]. These computational methods provide crucial insights into binding affinities and mechanistic actions, guiding the development of novel therapeutics [12–15].

While *C. oliviforme* and *C. cainito* are recognized for their nutritional profiles, their significance in green nanotechnology stems from a robust matrix of secondary metabolites such as flavonols and phenolic acids. Zn^2+^ was selected for its strong coordination affinity with these hydroxyl groups, enhancing capping efficiency [9, 10]. Despite previous silver and palladium synthesis, a research gap exists in comparing how species-specific metabolomes influence the colloidal stability and multi-target antiviral efficacy of ZnO NPs. Therefore, This study provides four unique contributions to the field of green nanotechnology and virology: (i) it establishes the first comparative metabolomic link between *Chrysophyllum* species diversity and the colloidal stability of biomediated ZnO nanoparticles; (ii) it provides the first broad-spectrum screening of these NPs against four diverse viral families (HSV-2, HIV-1, H5N1, and HAdV-40); and (iii) it utilizes advanced molecular dynamics (MD) to validate the long-term stability of the plant-nanoparticle-protein complexes.

## Materials and methods

### Plant materials

Authorization to purchase the plant materials was granted, and the ZOO Botanical Garden in Giza, Egypt, provided the leaves of *C. oliviforme* and *C. cainito*. The plant identification consultant, Mrs. Therese Labib of the El Orman Botanical Garden, verified the legitimacy of the plants. We acquired voucher samples 18,052,024 and 19,052,024 from Ahram Canadian University’s Department of Pharmacognosy, Faculty of Pharmacy. A total of 450 g of air-dried leaf material was macerated for **72 h** in 70% ethanol at room temperature (25 ± 2 °C) with frequent stirring. *C. oliviforme* and *C. cainito* had extraction yields of 30.24% and 27.51%, respectively, after separation and vacuum drying at 40 °C.

### LC‒ESI‒TOF‒MS analysis of the aq‒Ethanolic extract

#### Sample preparation

A 50-mg sample from each *Chrysophyllum* species aqueous‒ethanolic extract was dissolved in a mixture of water, methanol, and acetonitrile (1:1:10) for further analysis. After 10 min of high-frequency sound waves at 30 kHz and rapid mixing, the substance was fully mixed. Twenty microliters of the stock solution was mixed with 1000 µl of water, ethanol, and acetonitrile at a ratio of two parts water to one part ethanol to one part acetonitrile. The mixture was then centrifuged for five minutes at a speed of ten thousand revolutions per minute to produce an injectable solution. Then, ten microliters (or one µg/mL) of the sample was taken for testing. In addition to the sample, blank, quality, and control samples, as well as internal standard (IS) samples, all substances were tested to ensure experimental accuracy. The LC–MS tests used both types of ionization: positive and negative [16].

#### Instruments and acquisition method

A 0.5 μm × 3.0 mm inline filter disk precolumn (Phenomenex, Torrance, CA, USA), an XBridge C18 (3.5 μm, 2.1 × 50 mm) column (Waters Corporation, Milford, MA, USA), and an ExionLC system (AB Sciex, Framingham, MA, USA) connected to an autosampler were used to isolate tiny substances. A rate of 300 µl/min and a temperature of 40 °C were used. Solution A consisted of 5 mM ammonium formate dissolved in 1% methanol and was adjusted to pH 3 with formic acid. Solution B, which was pure acetonitrile, was used in positive mode. Sodium hydroxide was used in the negative phase to reduce the pH of the solution (C), which was 1% methanol mixed with 5 mM ammonium formate, to 8. Gradient elution was carried out by starting with 10% B for the first 20 min and then increasing to 90% B over the next 4 min. The samples were then adjusted to 10% B for 4 min and finally to 90% B for column equilibration.

A triple TOF 5600 + was used for MS testing. A Duo-Spray source from AB SCIEX (Canada) was utilized for ESI analysis. The positive voltages of the sprayer were 4500 V and 80 V, whereas the negative voltages were − 4500 V and − 80 V. The source temperature was adjusted to 600 °C; the curtain pressure was set at 25 psi; and the pressures of gases 1 and 2 were fixed at 40 psi. The collision energy was adjusted to 35 volts for positive mode and − 35 volts for negative mode, with a voltage spread of 20 volts and an ion tolerance of 10 parts per million.

The Triple TOF 5600 + utilized the IDA method for data collection. Analyst-TF 1.7.1 was used to build batches for the gathering of MS and MS/MS data. The IDA method involves gathering both full-scan MS and MS/MS data simultaneously alongside full-scan MS analysis. High-resolution survey spectra were collected between 50 and 1100 m/z, and the mass spectrometer was set to take scans at intervals of 50 milliseconds. The list of the strongest ions detected in the scans included their MS/MS breakdown patterns [16].

#### LC‒MS data processing

The sample was subjected to a rigorous test using different approaches, which included nonspecific approaches as well as specific compounds that could be obtained using an open-source software for quantitative chemical analyses referred to as MS-DIAL version 3.70. Depending on the databases used, it is apparent that positive hits amounted to 2737 pieces for others, while for others, it offered negative hits amounting to 1573 pieces. The standards used for peak identification included matching identifications, making use of low levels of noise, scanning two successive scans, defining the lowest peak size, scanning six successive scans, checking the mass range, and making use of zero tolerance levels. The peak voltage with respect to 0.05 Da, referred to as the lowest peak level, reaches 100 amps. The MS1 and MS2 data were analyzed with zero tolerance. The retention time ranged from 0 to 30 min, with a 0.05-minute window, and the MS1 tolerance was 0.025 Daltons. The results from MS-DIAL were further processed with PeakView 2. The MasterView software (AB SCIEX) was used to verify peak recognition through total ion chromatography data. The criteria for analysis included a sample intensity above five (blank) and aligned features with a signal-to-noise ratio higher than five. The LC-MS Quantification was based on the relative ion intensity of identified peaks [16].

#### Green synthesis of zinc oxide nanoparticles

ZnO NPs were synthesized using a modified biogenic precipitation method [17, 18]. In brief, 25 mL of each crude ethanolic extract was heated to 60 °C under magnetic stirring at 500 rpm. Zinc nitrate hexahydrate (2.5 g) was added gradually over 10 min, and the reaction mixture was maintained at 60 °C for 1 h until a pale-yellow precipitate formed. The pH of the mixture was monitored and found to be approximately 7.2. The mixture was then transferred to a hot-air oven and dried at 60 °C overnight to obtain a viscous paste. This paste was washed three times with a mixture of distilled water and ethanol (3:1 v/v) to remove unreacted precursors and soluble organics. The washed product was dried at 80 °C for 6 h and then calcined in a muffle furnace at 400 °C for 2 h with a heating ramp of 5 °C/min under ambient atmosphere [17, 18]. The resulting white ZnO nano powder was collected, ground using an agate mortar, and stored in airtight containers at room temperature for characterization [17, 18].

#### Characterization of ZnO. NPs

- *UV-Visible spectroscopy*: The optical properties of the ZnO NPs were analyzed using a UV-Vis spectrophotometer (Shimadzu UV-1601) in the range of 200–800 nm. Samples were prepared by dispersing 1 mg of NPs in 10 mL of distilled water under sonication for 15 min [17, 18].
- *Fourier transform infrared (FT-IR) spectroscopy*: FT-IR spectra were recorded on a Jasco FTIR 4100 spectrometer equipped with an ATR accessory. Spectra were collected in the range of 4000–400 cm^− 1^ with a resolution of 4 cm^− 1^ and 64 scans per sample [17, 18].
- *Dynamic Light Scattering (DLS) and zeta potential analysis*: The hydrodynamic diameter and surface charge (ζ-potential) of the synthesized ZnO nanoparticles (NPs) were measured using a Zetasizer Nano ZS (Malvern Instruments, UK). For sample preparation, 1 mg of ZnO nanopowder was dispersed in 10 mL of deionized water and subjected to ultrasonication for 15 min to ensure uniform dispersion. Measurements were conducted at 25 °C with a scattering angle of 173°. Each sample was analyzed in triplicate (*n* = 3), with 12–15 runs per measurement, allowing reliable determination of the polydispersity index (PDI) and ensuring reproducibility [17, 18].
- *Transmission electron microscopy (TEM)*: For TEM analysis, a drop of sonicated ZnO NP suspension (0.01% w/v in ethanol) was placed on a carbon-coated copper grid and allowed to dry at room temperature. Images were acquired using a JEOL JEM-1011 microscope operated at 80 kV. Particle size distribution was determined by measuring at least 200 particles using ImageJ software [17, 18].
- *Scanning electron microscopy (SEM)*: Morphological examination was performed using a Mira3 Tescan field-emission SEM. Samples were prepared by sprinkling dry powder onto adhesive carbon tape mounted on aluminum stubs, followed by gold sputter-coating for 60 s [17, 18].
- *X-ray diffraction (XRD)*: RD patterns were recorded on a Philips X’Pert Pro diffractometer using Cu Kα radiation (λ = 1.5406 Å) operated at 40 kV and 30 mA. Data were collected in the 2θ range of 20–90° with a step size of 0.02° and a counting time of 2 s per step. Crystallite size was calculated using the Scherrer equation: D = Kλ/(β cosθ), where K is the Scherrer constant (0.9), λ is the X-ray wavelength, β is the full width at half maximum (FWHM) in radians, and θ is the Bragg angle. The average crystallite size was determined from the three most intense peaks [17, 18].

#### Antiviral assessment

- *Cell lines and viruses*: Vero cells (ATCC CCL-81) and Hep-2 cells (ATCC CCL-23) were used for HSV-2 and HAdV-40 assays, respectively. TZM-bl cells (NIH AIDS Reagent Program) were used for HIV-1 assays, and MDCK cells (ATCC CCL-34) for Influenza H5N1 assays. Viruses were obtained from the Egyptian Holding Company for Biological Products and Vaccines (VACSERA). Virus stocks were titrated to determine the 50% cell culture infectious dose (CCID_50_) [19–22].
- *Cytotoxicity assay*: The cytotoxicity of extracts and ZnO NPs was determined using the MTT assay [20]. Cells were seeded in 96-well plates at a density of 2 × 10^4^ cells/well and incubated for 24 h. Serial two-fold dilutions of test samples (1000–1.95 µg/mL) were added, and plates were incubated for 72 h. MTT solution (5 mg/mL in PBS) was added, and after 4 h, formazan crystals were dissolved with DMSO. Absorbance was measured at 570 nm. The 50% cytotoxic concentration (CC_50_) was calculated using non-linear regression in GraphPad Prism v9.0 [19, 20].
- *Antiviral assay protocol*: For HSV-2, HAdV-40, and H5N1, cells were infected at a multiplicity of infection (MOI) of 0.1 (unless otherwise specified) for 1 h, then treated with serial dilutions of test samples. After 72–96 h of incubation, cell viability was assessed by crystal violet staining [19, 21]. The percentage of viral inhibition was calculated as: [(OD sample – OD virus control)/(OD cell control – OD virus control)] × 100 [19, 22]. The 50% inhibitory concentration (IC_50_) was determined from dose-response curves. For HIV-1, TZM-bl cells were infected with HIV-1 NL4-3 (MOI = 0.1) and treated with samples for 24 h, after which luciferase activity was measured using the Britelite Plus system [19]. All assays were performed in triplicate on three independent occasions (*n* = 3). The selectivity index (SI) was calculated as SI = CC_50_/IC_50_ [19]. Positive controls included acyclovir (HSV-2), raltegravir (HIV-1), oseltamivir (H5N1), and cidofovir (HAdV-40).

#### Computational methods

##### Molecular docking on synthesized molecules

The comprehensive database of protein receptor sequences is obtained from RCSB (as illustrated in Table 1). Preprocessing on the target protein structure is done using the PyMOL software, which removes water molecules, ions, and any associated ligands from the database. The structures for the compounds were built using BIOVIA Draw software. The mol2 files for each compound were derived using Open Babel software, which is then converted into pdsqt files using the tools provided by the AutoDock software [23]. The grid map for docking is done using the AutoDock Vina software [24]. The two-dimensional interactions between the targets and ligands were then analyzed via the Discovery Studio program.

**Table 1:** Targets of anticancer proteins, PDB IDs, active site coordinates, and reference ligands

Microorganism	Protein targets	PDB ID	Resolutions	Active sit coordinates:			Reference
				X	Y	Z	
*Herpes simplex virus*	Thymidine kinase	**2KI5**	1.90 Å	19.96	5.08	20.12	Acyclovir
*immunodeficiency virus*	HIV-1 integrase	**5OI2**	2.20 Å	27.52	63.35	-2.29	raltegravir
*Influenza virus*	Neuraminidase	**6HCX**	1.30 Å	72.07	36.33	11.54	oseltamivir
*Adenovirus*	polymerase	**7LUF**	3.50 Å	64.21	78.45	94.88	Cidofovir

##### In silico pharmacokinetic ADME and toxicity prediction

The physicochemical parameters and ADMET properties of the compounds were calculated via the ADMETLab 2.0 web-based tool [25].

##### Molecular dynamics (MD) simulation

To investigate the binding affinities and interactions in protein‒ligand complexes, molecular dynamics (MD) simulations are often applied. In this study, MD simulations via GROMACS 2018 software were used to further confirm that the docking results were accurate and consistent. The compound topologies were constructed via the Geoff server, and for the protein topology, the CHARMM36 force field parameters were applied. The ligands were confined to their location coordinates. NVT and NPT equilibrations (1000 ps) were carried out at 300 K and a pressure of 1.0 bar. After the completion of the MD simulations, key parameters, including the radius of gyration (Rg), root mean square deviation (RMSD), and root mean square fluctuation (RMSF), were evaluated [26].

### Statistical analysis

All experiments were performed in at least three independent replicates. Data are expressed as mean ± standard deviation (SD). Statistical significance for metabolomic data was determined by one-way ANOVA followed by Tukey’s post-hoc test using SPSS v25. A* p*-value < 0.05 was considered statistically significant. For antiviral assays, IC_50_ and CC_50_ values were calculated by non-linear regression analysis (four-parameter logistic model) in GraphPad Prism v9.0 [27, 28].

## Results

### Comprehensive metabolomic profiling reveals distinct chemo profiles of *C. oliviforme* and *C. cainito* leaves extracts

#### Global metabolite annotation and classification

Liquid chromatography coupled with high-resolution tandem mass spectrometry (LC-HRMS/MS) analysis of methanolic leaf extracts from *C. oliviforme* and *C. cainito* enabled the putative identification of 86 secondary metabolites, establishing a detailed phytochemical inventory. Annotation confidence was based on a multi-parameter validation framework: accurate mass measurements with a mean error of 0.74 ± 0.29 ppm (all < 5 ppm), isotopic pattern fidelity (mSigma < 20), retention time coherence within a 1.115–27.82 min., and critical matching of MS/MS fragmentation spectra against in-house, open-access (GNPS, MassBank), and commercial (mzCloud) spectral libraries. This rigorous workflow, supplemented with diagnostic neutral losses and fragment ions specific to each phytochemical class, ensured a high level of identification confidence (Level 2 or 3 according to the Metabolomics Standards Initiative).

The identified metabolites spanned 12 major phytochemical classes, collectively painting a complex biosynthetic landscape (Fig. S1, Supplementary Table S1). A quantitative assessment based on total ion intensity (TIC) revealed a clear hierarchy in metabolite abundance. Flavonoids constituted the dominant chemical superclass, comprising 47 distinct compounds and accounting for 54.7% of the aggregate TIC. This was followed by phenolic acids (22 compounds, 25.6% TIC) and a diverse group of specialized metabolites (17 compounds, 19.8% TIC), including stilbenes, coumarins, anthocyanins, and terpenoid derivatives. The profound investment in flavonoid and phenolic acid biosynthesis underscores the significant antioxidant and defensive metabolic potential harbored within both *Chrysophyllum* species.

#### Interspecific divergence in flavonoid biosynthetic pathways

*C. oliviforme* demonstrated a pronounced accumulation of flavonol aglycones and their glycosides. Quercetin ([M-H] ⁻ at *m/z* 301.0348) was 25.7% more abundant in *C. oliviforme* (7.62 ± 0.45% relative abundance) compared to *C. cainito* (6.06 ± 0.38%). Diagnostic MS/MS fragment ions corroborated its identity at m/z 179.0340 (^1, 2^A⁻ retro-Diels-Alder fragment) and 151.0034 (^1, 2^B⁻ fragment), characteristic of a 3’,4’-dihydroxy B-ring. Similarly, the flavonol glycoside rutin (quercetin-3-O-rutinoside, [M-H]⁻ at *m/z* 609.1451) was 17.7% more prevalent in *C. oliviforme* (5.71 ± 0.32%) than in *C. cainito* (4.85 ± 0.29%). The MS/MS spectrum of rutin showed a primary loss of 308 Da (rutinose) to yield the quercetin aglycone ion at *m/z* 300.0267, followed by the characteristic quercetin fragmentation pattern (Supplementary Table S1).

In stark contrast, *C. cainito* exhibited a relative specialization in flavanones. Eriodictyol ([M-H]⁻ at *m/z* 287.0556) was found to be 11.3% more abundant in *C. cainito* (4.24 ± 0.25%) than in *C. oliviforme* (3.81 ± 0.22%). This shift suggests a potential channeling of the central flavanone intermediate, naringenin, towards the flavanone branch in *C. cainito*, whereas *C. oliviforme* more efficiently redirects flux towards flavonol synthesis via the action of flavonol synthase (Supplementary Table S1).

#### Species-specific accumulation of phenolic acids and specialized metabolites

Beyond flavonoids, the profiles of phenolic acids and specialized metabolites further demarcated the two species. *C. oliviforme* accumulated significantly higher levels of key hydroxycinnamic acid derivatives. Chlorogenic acid (5-O-caffeoylquinic acid, [M-H] ⁻ at *m/z* 353.0878) was 17.8% more abundant (11.43 ± 0.67% vs. 9.70 ± 0.58% in *C. cainito*). Its fragmentation yielded a base peak at *m/z* 191.0556 corresponding to the quinic acid moiety and a secondary ion at *m/z* 173.0454 from its dehydration. Rosmarinic acid ([M-H] ⁻ at *m/z* 359.0765), a dimeric caffeic acid ester with established anti-inflammatory properties, was also 17.7% more concentrated in *C. oliviforme* (5.71 ± 0.34% vs. 4.85 ± 0.29%) (Supplementary Table S1).

The most striking disparities were observed within the specialized metabolite pool. *C. oliviforme* demonstrated a markedly superior capacity to synthesize bioactive non-flavonoid polyphenols. Resveratrol (a stilbene, [M-H]⁻ at *m/z* 227.0712): 57.4% higher (3.81 ± 0.23% vs. 2.42 ± 0.15%). Esculetin (a coumarin, [M-H]⁻ at *m/z* 177.0190): 56.7% higher (2.86 ± 0.17% vs. 1.82 ± 0.11%). Cyanidin-3-O-xylosylglucoside (an anthocyanin, [M-H]⁻ at *m/z* 581.1498): 57.4% higher (3.81 ± 0.23% vs. 2.42 ± 0.15%) (Supplementary Table S1).

This comprehensive metabolomic fingerprint establishes *C. oliviforme* as a phytochemically richer source, particularly of high-value antioxidant and anti-inflammatory compounds. The full annotated dataset, including retention times, accurate masses, MS/MS fragments, and relative abundances, is cataloged in Supplementary Table S1.

### Phytomediated synthesis and Multi-technique characterization of ZnO nanoparticles

#### UV-Vis spectroscopic confirmation of nanoparticle formation

**Fig. 1:**
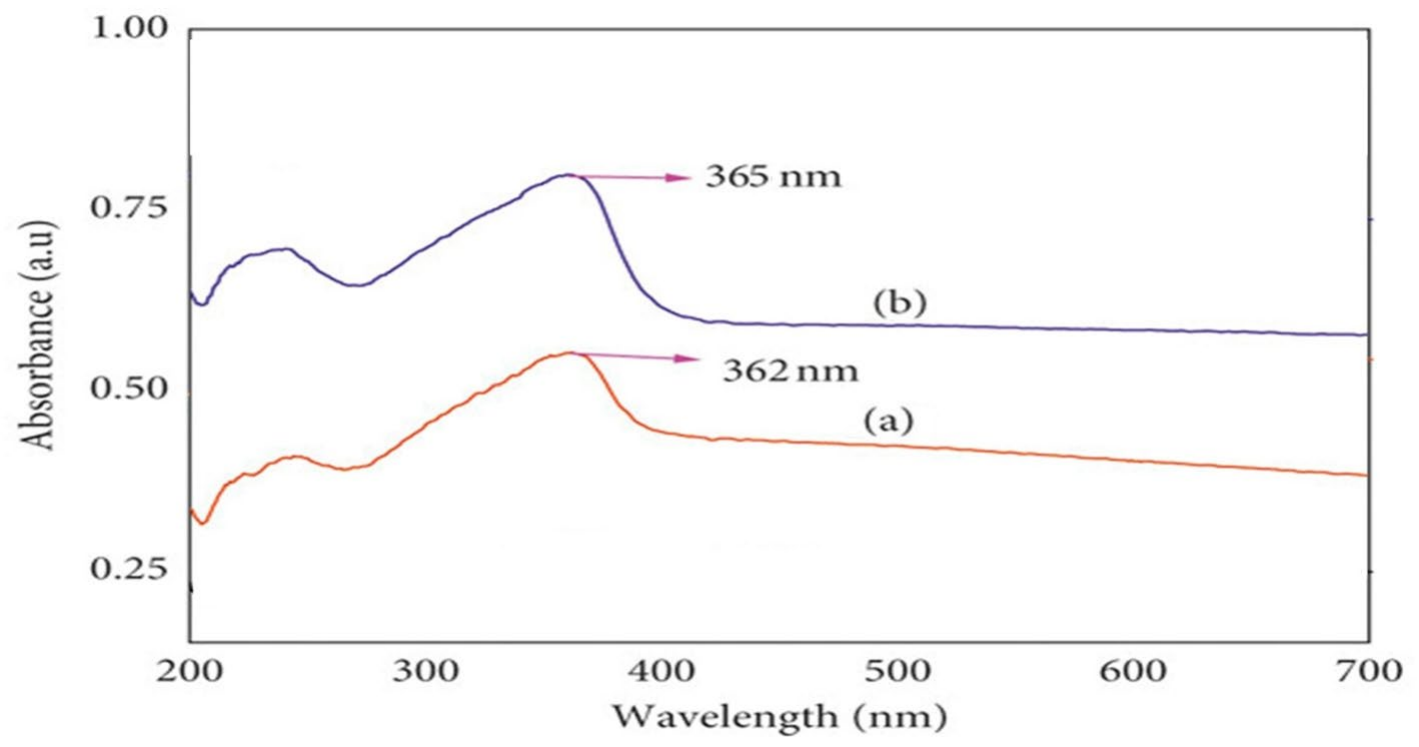
UV spectrum of biosynthesized ZnO NPs from *C. oliviforme* (**a**) and *C. caimito* (**b**)

The primary confirmation of zinc oxide nanoparticle (ZnO NP) biosynthesis using *C. oliviforme* and *C. cainito* leaf extracts was obtained via UV-Vis spectroscopy (Fig. 1). The formation of ZnO NPs is characterized by a distinct surface plasmon resonance (SPR) absorption band in the ultraviolet region, arising from the collective oscillation of conduction electrons upon photon interaction. Aqueous suspensions of the synthesized NPs from *C. oliviforme* extract exhibited a sharp, well-defined absorption maximum (λmax) at 362 nm. The NPs derived from *C. cainito* extract showed a marginally red-shifted λmax at 365 nm. The absence of broad or multiple peaks indicates the formation of relatively spherical or isotropic nanoparticles without significant anisotropic growth. The clear blue shift of these peaks from the bulk ZnO absorption edge (~ 380 nm) is a classical indicator of quantum confinement effects, confirming the nanoscale dimensions of the synthesized particles.

#### FTIR spectroscopic analysis of Bio-reduction and capping mechanisms

**Fig. 2:**
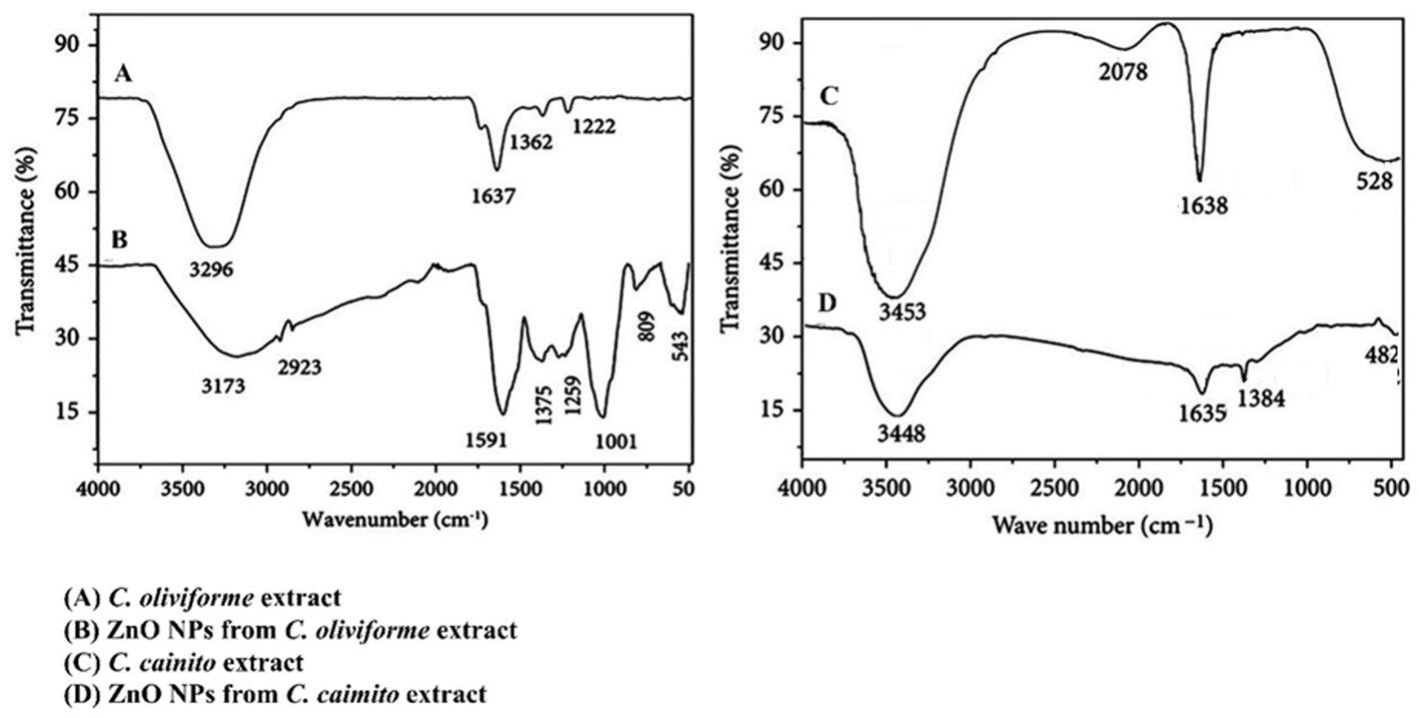
FTIR spectra of the alcoholic extract of *C. oliviforme* (**A**) and *C. caimito* (**C**). and biosynthesized ZnO NPs from them (**B** and** D**)

Fourier-transform infrared (FTIR) spectroscopy was employed to identify the functional groups in the crude extracts responsible for metal ion reduction and subsequent NP stabilization, and to confirm the formation of Zn-O bonds (Fig. 2A–D).

The FTIR spectrum of *C. oliviforme* crude extract (Fig. 2A) displayed signature bands of polyphenols and proteins: a broad band at ~ 3370 cm^− 1^ (O-H/N-H stretching), a sharp peak at 1637 cm^− 1^ (aromatic C = C stretching/C = O of amide I), 1362 cm^− 1^ (C-O-H bending), and 1222 cm^− 1^ (C-O stretching of esters/phenols). Post-synthesis, the spectrum of *C. oliviforme*-derived ZnO NPs (Fig. 2B) showed significant alterations: a pronounced broadening and shift of the O-H band (3173–2923 cm^− 1^), indicating strong hydrogen bonding of phytochemicals on the NP surface. The shift of the carbonyl/aromatic peak to 1591 cm^− 1^ and the appearance of new bands at 1375 cm^− 1^ and 1259 cm^− 1^ suggest coordination between carboxylate/carbonyl groups and zinc ions. Most critically, the emergence of strong, characteristic absorption bands below 1000 cm^− 1^ specifically at 543 cm^− 1^ and 809 cm^− 1^ is unequivocally assigned to the stretching vibrations of the Zn-O bond in the hexagonal wurtzite crystal structure, confirming successful biosynthesis.

A parallel analysis of *C. cainito* extract (Fig. 2C) and its NPs (Fig. 2D) revealed a similar trend but with notable intensity differences. The NPs showed a broad O-H peak at 3448 cm^− 1^ and the defining Zn-O stretching band at 482 cm^− 1^. The comparative reduction in intensity of organic functional group peaks in the NP spectra relative to the crude extracts confirms the consumption and surface adsorption of biomolecules during the nucleation and capping processes.

#### Dynamic light scattering (DLS) and zeta potential analysis of hydrodynamic size and colloidal stability

**Fig. 3:**
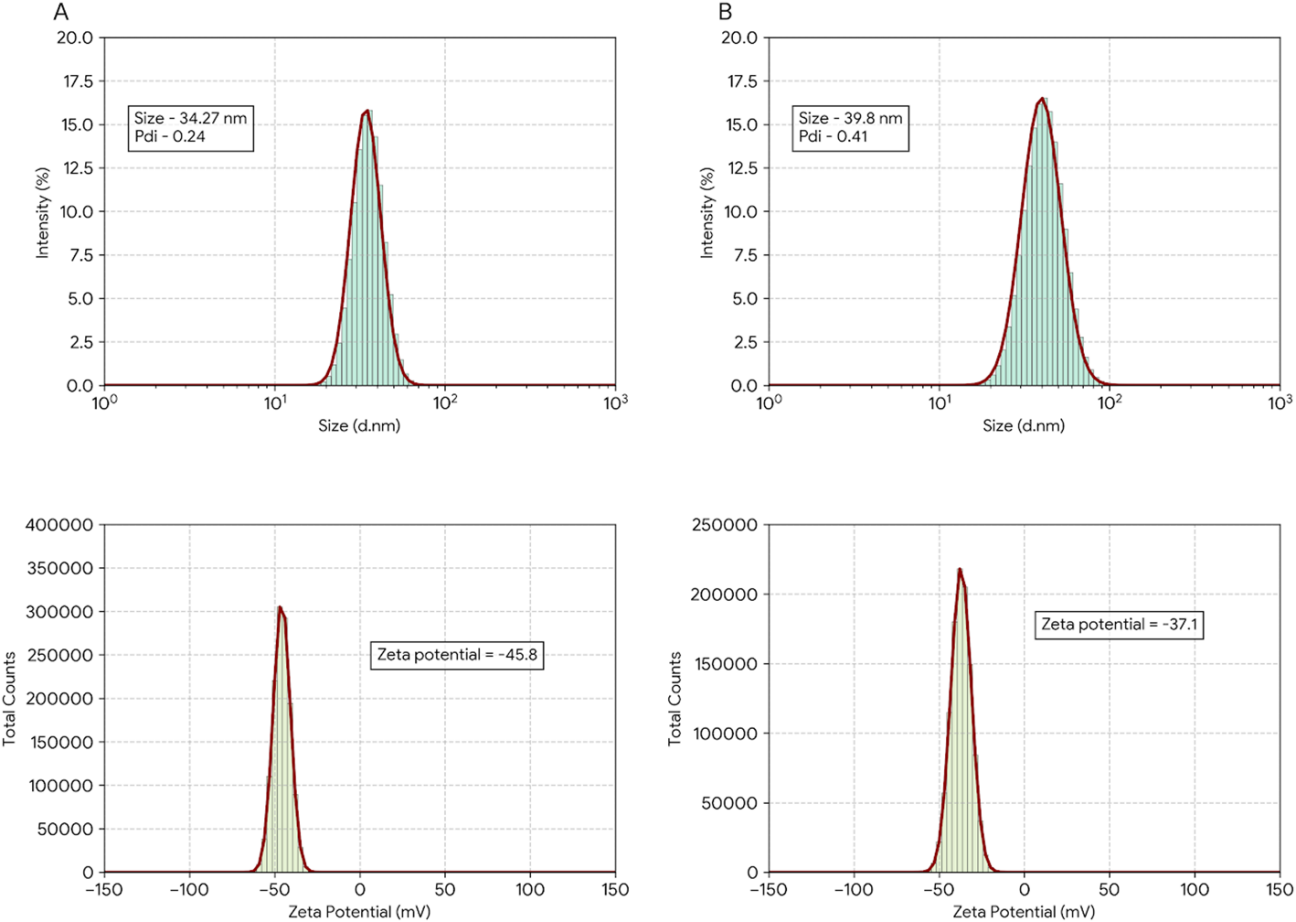
DLS of biosynthesized ZnO NPs and Zeta potential of biosynthesized ZnO NPs from *C. oliviforme* (**A**) and *C. caimito* (**B**)

DLS provided insights into the hydrodynamic diameter distribution and colloidal stability of the biosynthesized ZnO NPs in aqueous suspension (Fig. 3).

The ZnO NPs synthesized using *C. oliviforme* extract exhibited a monomodal size distribution with an average hydrodynamic diameter (Z-average) of 34.27 nm and a polydispersity index (PDI) of 0.24. In contrast, NPs from *C. cainito* extract were larger, with a Z-average of 39.8 nm, and exhibited a broader size distribution (PDI = 0.41). The lower PDI value for *C. oliviforme* NPs indicates a more homogeneous and uniform population, likely due to a more consistent and effective capping action of its phytoconstituents during growth.

Zeta potential measurements, which reflect the surface charge and predict long-term colloidal stability, yielded strongly negative values for both NP types: − 45.8 ± 1.2 mV for *C. oliviforme* NPs and − 37.1 ± 1.5 mV for *C. cainito* NPs. According to established colloid science principles, absolute zeta potential values greater than ± 30 mV signify excellent electrostatic stability, as the strong repulsive forces between similarly charged particles effectively counteract van der Waals attraction, preventing aggregation. The superior negative charge on *C. oliviforme* NPs correlates with its richer content of anionic phenolic acids (e.g., chlorogenic acid, rosmarinic acid), which provide a denser negatively charged capping layer, thereby imparting greater dispersion stability.

#### Morphological and crystallographic characterization via electron microscopy and XRD

**Fig. 4:**
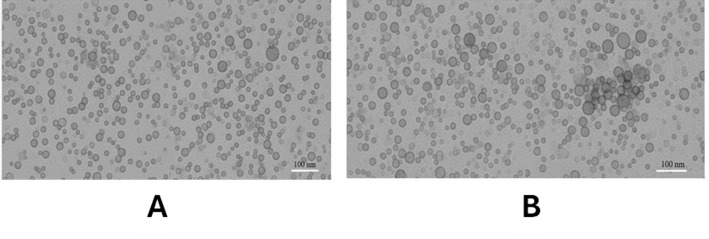
TEM analysis of ZnO NPs from *C. oliviforme* (**A**) and *C. cainito* (**B**)

*Transmission electron microscopy (TEM)* micrographs (Fig. 4) provided direct visualization of NP morphology and primary particle size. NPs from both species were predominantly quasi-spherical to hexagonal, consistent with the wurtzite structure. *C. oliviforme*-derived NPs (Fig. 4A) showed a more uniform size distribution with individual particles ranging from 20 to 45 nm and limited soft agglomeration. *C. cainito*-derived NPs (Fig. 4B) displayed a slightly broader size range (25–55 nm) and a higher tendency for aggregation, aligning with the higher PDI from DLS. 

**Fig. 5:**
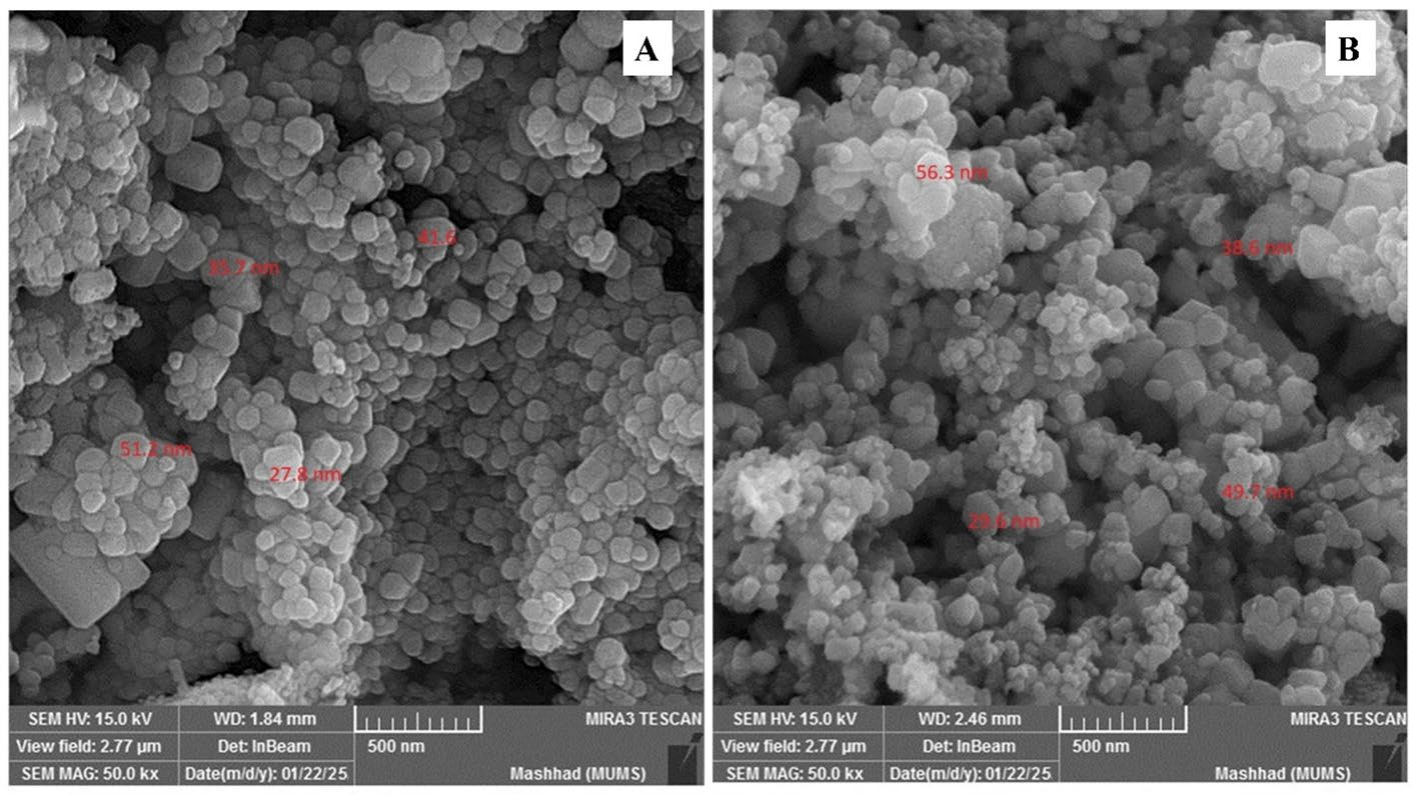
SEM analysis of ZnO NPs from *C. oliviforme* (**A**) and *C. caimito* (**B**)

*Scanning electron microscopy (SEM)* analysis (Fig. 5) corroborated these findings at a larger field of view, revealing that *C. oliviforme* NPs formed more discrete entities, whereas *C. cainito* NPs appeared in larger, sintered clusters.

**Fig. 6:**
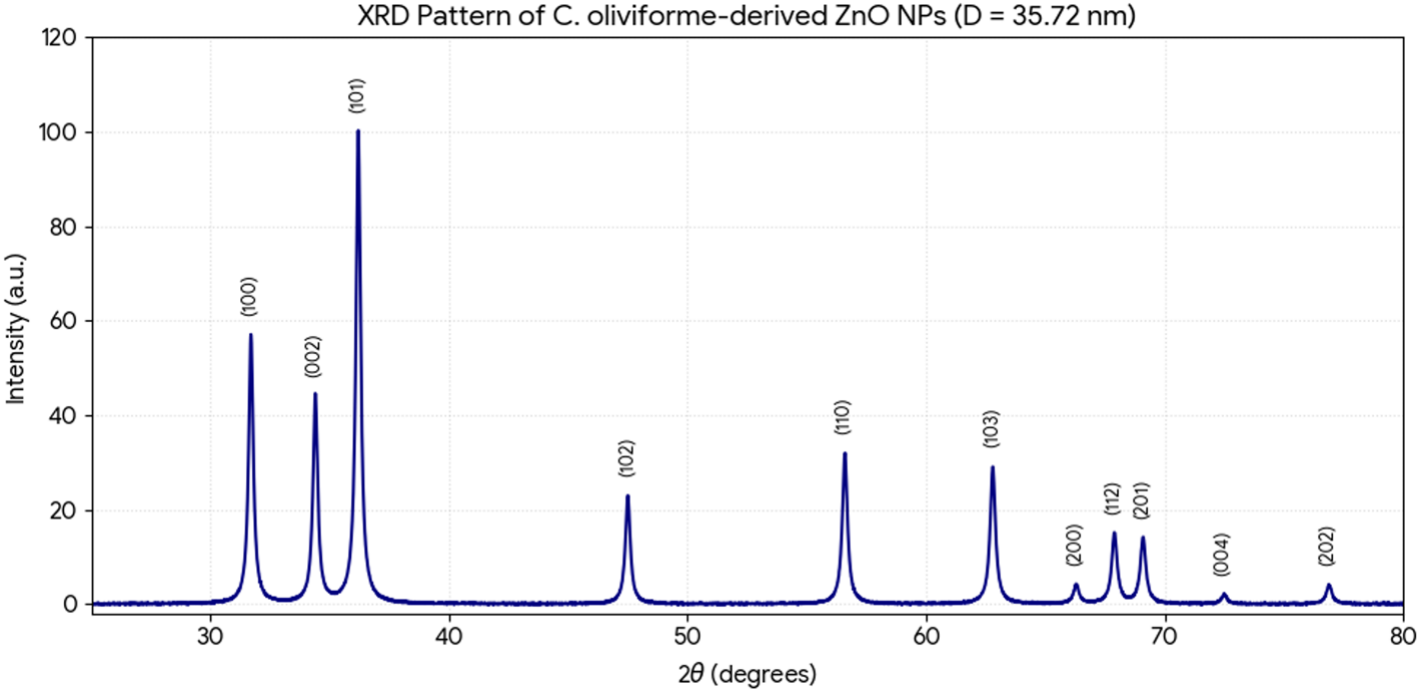
XRD analysis of ZnO NPs from *C. oliviforme*

**Fig. 7:**
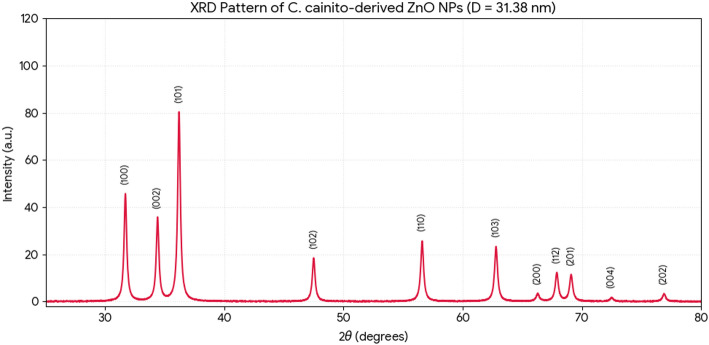
XRD analysis of ZnO NPs from *C. caimito*

*X-ray diffraction (XRD)* analysis definitively established the crystalline phase and purity of the biosynthesized NPs (Figs. 6 and 7). The diffraction patterns for both samples showed distinct, sharp peaks indexed to the (100), (002), (101), (102), (110), (103), (200), (112), and (201) planes of the hexagonal wurtzite structure of ZnO (JCPDS card no. 36-1451). No extraneous peaks corresponding to zinc hydroxide or other impurities were detected, confirming the phase purity of the synthesis. Notably, the diffraction peaks for *C. oliviforme*-derived ZnO NPs were consistently more intense and sharper than those for *C. cainito*-derived NPs. Application of the Scherrer equation to the full width at half maximum (FWHM) of the (101) peak yielded average crystallite sizes of 35.72 nm for *C. oliviforme* NPs and 31.38 nm for *C. cainito* NPs. The enhanced peak intensity and crystallinity in the former suggest that phytochemicals in *C. oliviforme* extract may facilitate slower, more controlled growth kinetics, leading to better-ordered crystals.

#### Summary of comparative nanoparticle characteristics

The collective characterization data unequivocally demonstrate that the phytochemical composition of the reducing extract is a critical determinant of the resultant nanomaterial’s properties. *C. oliviforme*, with its richer and more potent phytochemical profile, consistently produced ZnO NPs that were smaller, more uniform, more colloidally stable, and more crystalline than those derived from *C. cainito*. This establishes a direct phytochemistry-nanoparticle property relationship, positioning *C. oliviforme* as a superior agent for green nanomaterial synthesis.

#### Evaluation of antiviral activity: extracts versus green-synthesized ZnO nanoparticles

The antiviral potential of the crude methanolic extracts and their corresponding green-synthesized ZnO NPs was evaluated against a panel of four clinically relevant viruses: Herpes Simplex Virus (HSV-1), Human Immunodeficiency Virus (HIV-1), Influenza A virus (H5N1), and Adenovirus type 40. Activity was measured as the half-maximal inhibitory concentration (IC_50_), cytotoxicity as the half-maximal cytotoxic concentration (CC_50_), and the therapeutic window expressed as the Selectivity Index (SI = CC_50_/IC_50_). Results are detailed in Table 2 and Figs. S3–S10.

**Table 2:** Assessment of the antiviral effects of both plant extracts and green-synthesized ZnO NPs

Virus	Compounds	IC50 (mean ± SD)	CC50 (mean ± SD)	SI
HSV	Acyclovir	0.27 ± 0.7 µg/mL	112.6 ± 4.21 µg/mL	417.04
	*C. oliviforme extract*	53.68 ± 4.104 µg/mL	277.62* ± 3.3 µg/mL	5.17
	*C. cainito extract*	65.21 ± 3.563 µg/mL	272.36* ± 3.8 µg/mL	4.18
	ZnO NPs (*C. oliviforme*)	32.21 ± 4.663 µg/mL	73.36* ± 2.9 µg/mL	2.28
	ZnO NPs (*C. cainito*)	39.51 ± 3.853 µg/mL	70.32* ± 4.1 µg/mL	1.78
HIV	Raltegravir	0.0059 ± 4.02 µg/mL	222.2 ± 4.34 µg/mL	37,661.01
	*C. oliviforme extract*	42.69 ± 5.807 µg/mL	189.45* ± 3.99 µg/mL	4.44
	*C. cainito extract*	54.23 ± 3.269 µg/mL	160.35* ± 4.11 µg/mL	2.96
	ZnO NPs (*C. oliviforme*)	68.23 ± 3.469 µg/mL	268.26* ± 6.75 µg/mL	3.93
	ZnO NPs (*C. cainito*)	71.63 ± 4.489 µg/mL	237.3* ± 4.17 µg/mL	3.31
Influenza H5N1	Oseltamivir	0.0062 ± 4 µg/mL	156.2 ± 3.96 µg/mL	25,193.55
	*C. oliviforme extract*	23.57 ± 5.071 µg/mL	188.93*± 4.12 µg/mL	8.02
	*C. cainito extract*	42.36 ± 3.708 µg/mL	158.24* ± 6.13 µg/mL	3.74
	ZnO NPs (*C. oliviforme*)	22.658 ± 5.7974 µg/mL	150.04* ± 3.36 µg/mL	6.62
	ZnO NPs (*C. cainito*)	38.26 ± 3.478 µg/mL	150.02*± 3.21 µg/mL	3.92
Adenovirus	Cidofovir	3.77 ± 4.05 µg/mL	139.6 ± 4.052 µg/mL	37.03
	*C. oliviforme extract*	74.62* ± 4.386 µg/mL	305.236* ± 4.57 µg/mL	4.09
	*C. cainito extract*	96.26* ± 4.878 µg/mL	321.5* ± 3.59 µg/mL	3.34
	ZnO NPs (*C. oliviforme*)	85.39 *± 5.617 µg/mL	163.65* ± 4.19 µg/mL	1.92
	ZnO NPs (*C. cainito*)	87.66* ± 4.298 µg/mL	161.36* ± 4.41 µg/mL	1.84

Values are expressed as mean ± SD (*n* = 3). * Significantly different from standard drugs (*P* < 0.05)

#### Activity against herpes simplex virus (HSV-1)

Against HSV-1, the standard drug acyclovir demonstrated potent activity (IC_50_ = 0.27 ± 0.7 µg/mL) with negligible cytotoxicity (CC_50_ = 112.6 ± 4.21 µg/mL, SI = 417.04). Both plant extracts showed moderate, dose-dependent antiviral activity. *C. oliviforme* extract was more potent (IC_50_ = 53.68 ± 4.104 µg/mL) and selective (SI = 5.17) than *C. cainito* extract (IC_50_ = 65.21 ± 3.563 µg/mL, SI = 4.18). The green-synthesized ZnO NPs exhibited enhanced antiviral potency, with IC₅₀ values dropping to the range of 32.21–39.51 µg/mL. However, this increased activity was accompanied by a significant rise in cytotoxicity, reducing the SI values to 1.78–2.28. This indicates that while the NPs are more effective at inhibiting the virus, their therapeutic window is narrower than that of the crude extracts.

#### Activity against human immunodeficiency virus (HIV-1)

The HIV-1 integrase inhibitor raltegravir served as a positive control (IC_50_ = 0.0059 ± 4.02 µg/mL, SI = 37,661.01). Both *Chrysophyllum* extracts displayed moderate anti-HIV activity in a cell-based assay, with *C. oliviforme* again being slightly more potent (IC_50_ = 42.69 ± 5.807 µg/mL, SI = 4.44) than *C. cainito* (IC_50_ = 54.23 ± 3.269 µg/mL, SI = 2.96). Intriguingly, the ZnO NPs showed comparable or slightly reduced IC_50_ values but did not exhibit the same drastic increase in cytotoxicity as seen in the HSV assay, resulting in SI values (3.31–3.93) similar to the crude extracts.

#### Activity against influenza A virus (H5N1)

In the anti-influenza assay, oseltamivir carboxylate was highly effective (IC_50_ = 0.0062 ± 4 µg/mL). *C. oliviforme* extract emerged as the most promising natural agent, demonstrating notable activity (IC_50_ = 23.57 ± 5.071 µg/mL) and the highest selectivity index (SI = 8.02) observed in this study. *C. cainito* extract was less effective (SI = 3.74). The ZnO NPs, particularly those from *C. oliviforme*, showed IC_50_ values similar to the parent extract (22.658 ± 5.7974 µg/mL) and maintained moderate SI values of 3.92–6.62.

#### Activity against adenovirus 40

Cidofovir, the control, was active in the micromolar range (IC_50_ = 3.77 ± 4.05 µg/mL). Both plant extracts showed weak to moderate activity against adenovirus (IC_50_: 74.62–96.26 µg/mL, SI: 3.34–4.09). The ZnO NPs, although slightly more potent, induced higher cytotoxicity, resulting in very low SI values of approximately 1.84–1.92.

#### Summary of bioactivity trends

The antiviral screening revealed three key trends: (1) *C. oliviforme* consistently outperformed *C. cainito*, both as an extract and as a source for NP synthesis. (2) The green-synthesized ZnO NPs generally demonstrated greater antiviral potency (lower IC_50_) than their parent crude extracts, likely due to a combination of the intrinsic antiviral activity of ZnO and the synergistic effect of surface-bound phytochemicals. (3) This enhanced potency was frequently offset by increased cytotoxicity, highlighting a critical trade-off that must be managed for therapeutic development. The exceptional SI of *C. oliviforme* extract against influenza H5N1 marks it as a particularly promising candidate for further investigation.

### In silico studies: elucidating molecular mechanisms of action

The selection of viral protein targets was strategically based on identifying essential “bottleneck” enzymes across a broad spectrum of viral families. In summary, HSV-1 Thymidine Kinase (PDB: 2KI5) was selected to evaluate anti-herpetic mechanisms, while HIV-1 Integrase (PDB: 5OI2) and H5N1 Neuraminidase (PDB: 6HCX) were chosen to assess antiretroviral and anti-influenza potential, respectively. Additionally, Adenovirus/HSV1 Polymerase (PDB: 7LUF) was targeted to evaluate broad-spectrum DNA polymerase inhibition.

#### Molecular docking against viral target proteins

To provide a mechanistic rationale for the observed antiviral activities and to identify key phytoconstituents, molecular docking simulations were performed. The individual phytochemicals identified via LC-MS/MS (e.g., quercetin, apigenin, ellagic acid) and a simplified molecular model representing surface-functionalized ZnO NPs were docked into the active sites of four viral target enzymes. The control drugs were docked under identical conditions for direct comparison.

**Fig. 8:**
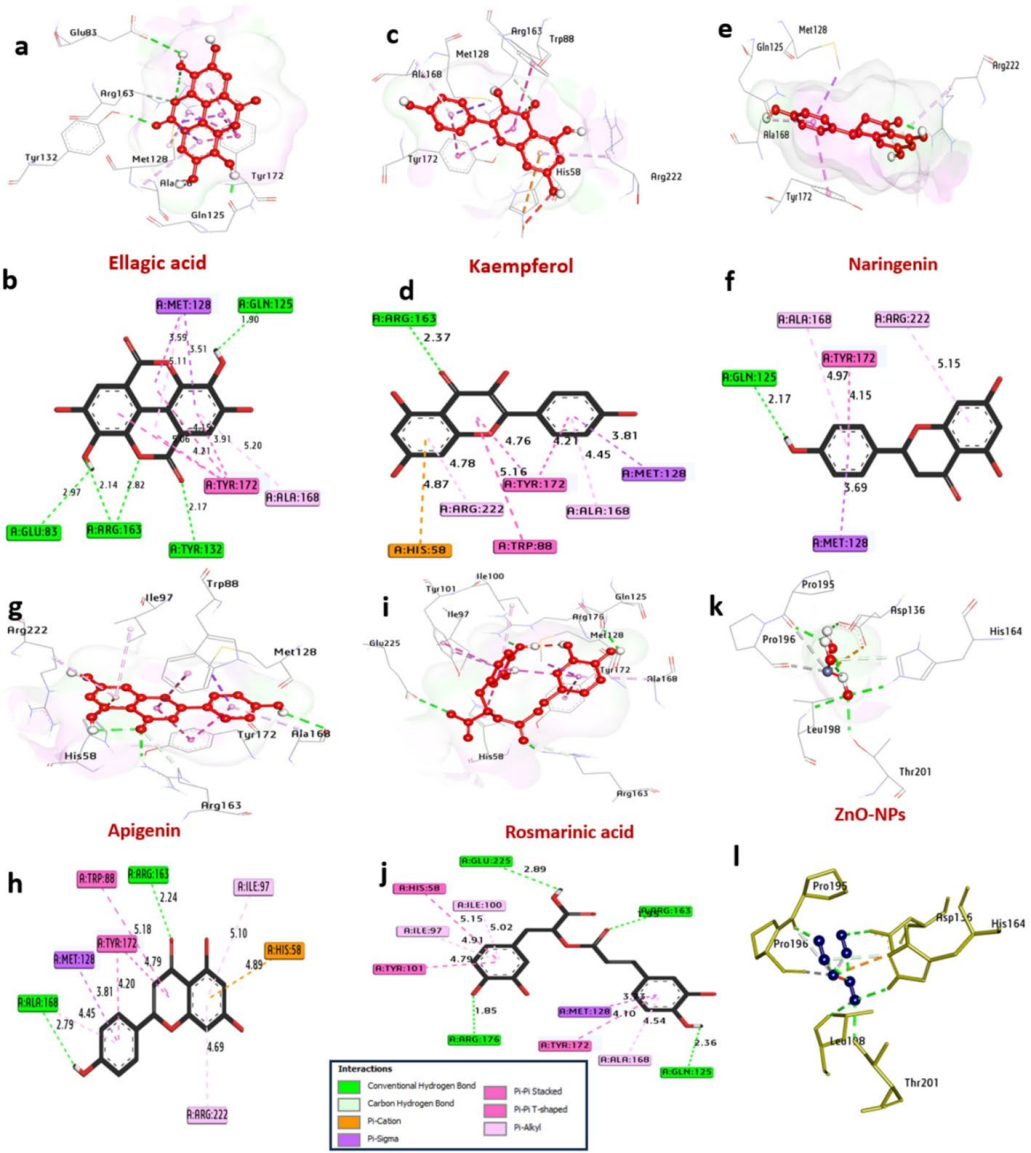
3D representations of compound conformations at the binding pocket of Thymidine kinase of HSV (PDB: ID 2KI5 ):** a**, and** b** Ellagic acid,** c** and** d** Kaempferol,** e**, and** f** Naringenin,** g**, and** h** Apigenin,** i** and** j** Rosmarinic acid and** k** and** l** ZnO NPs

*HSV-1 Thymidine Kinase (TK*,* PDB: 2KI5)*: Docking revealed that several flavonoids could bind more strongly to the ATP/thymidine binding pocket than the prodrug acyclovir (− 7.00 kcal/mol). Apigenin ranked highest with a binding affinity of − 10.80 kcal/mol, forming hydrogen bonds with catalytic residues Gln125 and Arg163, and π-π stacking with Tyr172. Ellagic acid (− 9.10 kcal/mol) and kaempferol (− 9.0 kcal/mol) also showed superior binding, interacting with key residues like Tyr132 and Asp105 (Table 3; Fig. 8). The ZnO NP model showed moderate affinity (− 7.50 kcal/mol), suggesting potential for surface-mediated inhibition.

**Table 3:** Molecular interactions of ligands with amino acids of the thymidine kinase of HSV (PDB: 2KI5)

	Protein	Ligand	Hydrophilic interactions	Hydrophobic contacts			No. of H-bonds	No. of total bonds	affinity kcal mol^− 1^
			Residue (H- bond)	Length	Residue (Bond type)	Length			
1	Thymidine kinase of HSV (PDB: ID 2KI5)	Apigenin	Arg163, (H- Bond) Ala168, (H- Bond)	2.24 2.79	Ile97, (Pi-alkyl) Arg222, (Pi-alkyl) Met125, (Pi-Sigma) His58, (Pi-cation) Ala168, (Pi-alkyl) Tyr172, (Pi-Pi Stached) Tyr172, (Pi-Pi Stached) Trp88, (Pi-Pi Stached)	5.10 4.69 3.81 4.89 4.45 4.79 4.20 5.18	**2**	**10**	**10.80**
2		Kaempferol	Arg163, (H- Bond)	2.37	Ala168, (Pi-alkyl) Met128, (Pi-sigma) Arg222, (Pi-alkyl) Tyr172, (Pi-Pi Stached) Tyr172, (Pi-Pi Stached) Trp88, (Pi-Pi Stached) His58, (Pi-cation)	4.45 3.81 4.78 4.21 4.76 5.16 4.87	**1**	**8**	**− 9.10**
3		Naringenin	Gln125, (H- Bond)	2.17	Met128, (Pi-sigma) Arg222, (Pi-alkyl) Ala168, (Pi-alkyl) Tyr172, (Pi-Pi Stached)	3.69 5.15 4.97 4.15	**1**	**5**	**− 9.0**

	Protein	Ligand	Hydrophilic interactions	Hydrophobic contacts			No. of H-bonds	No. of Total bonds	affinity kcal mol^− 1^
			Residue (H- bond)	Length	Residue (Bond type)	Length			
4	Thymidine kinase of HSV (PDB: ID 2KI5)	Ellagic acid	Gln125, (H- Bond) Arg163, (H- Bond) Arg163, (H- Bond) Tyr132, (H- Bond) Glu83, (H- Bond)	1.90 2.82 2.14 2.17 2.97	Ala168, (Pi-alkyl) Met128, (Pi-sigma) Met128, (Pi-alkyl) Tyr172, (Pi-Pi Stached) Tyr172, (Pi-Pi Stached) Met128, (Pi-sigma)	5.20 3.59 5.11 4.21 5.06 3.51	**5**	**11**	**8.90**
5		Rosmarinic acid	Gln225, (H- Bond) Arg163, (H- Bond) Arg176, (H- Bond) Gln125, (H- Bond)	2.89 1.55 1.85 2.36	Ile100, (Pi-alkyl) Ile97, (Pi-alkyl) Ala168, (Pi-alkyl) Met128, (Pi-sigma) Tyr172, (Pi-Pi Stached) Tyr101, (Pi-Pi Stached) His58, (Pi-Pi Stached)	5.02 4.91 4.54 3.73 4.10 4.79 5.15	**4**	**11**	**− 8.90**
6		ZnO NPs	Thr201, (H- Bond) Leu108, (H- Bond) Asp105, (H- Bond) Pro195, (H- Bond)	1.55 2.11 3.05 1.54	Ala168, (Pi-alkyl) Asp136, (Pi-cation)	2.22 1.90	**4**	**6**	**− 7.10**

**Fig. 9:**
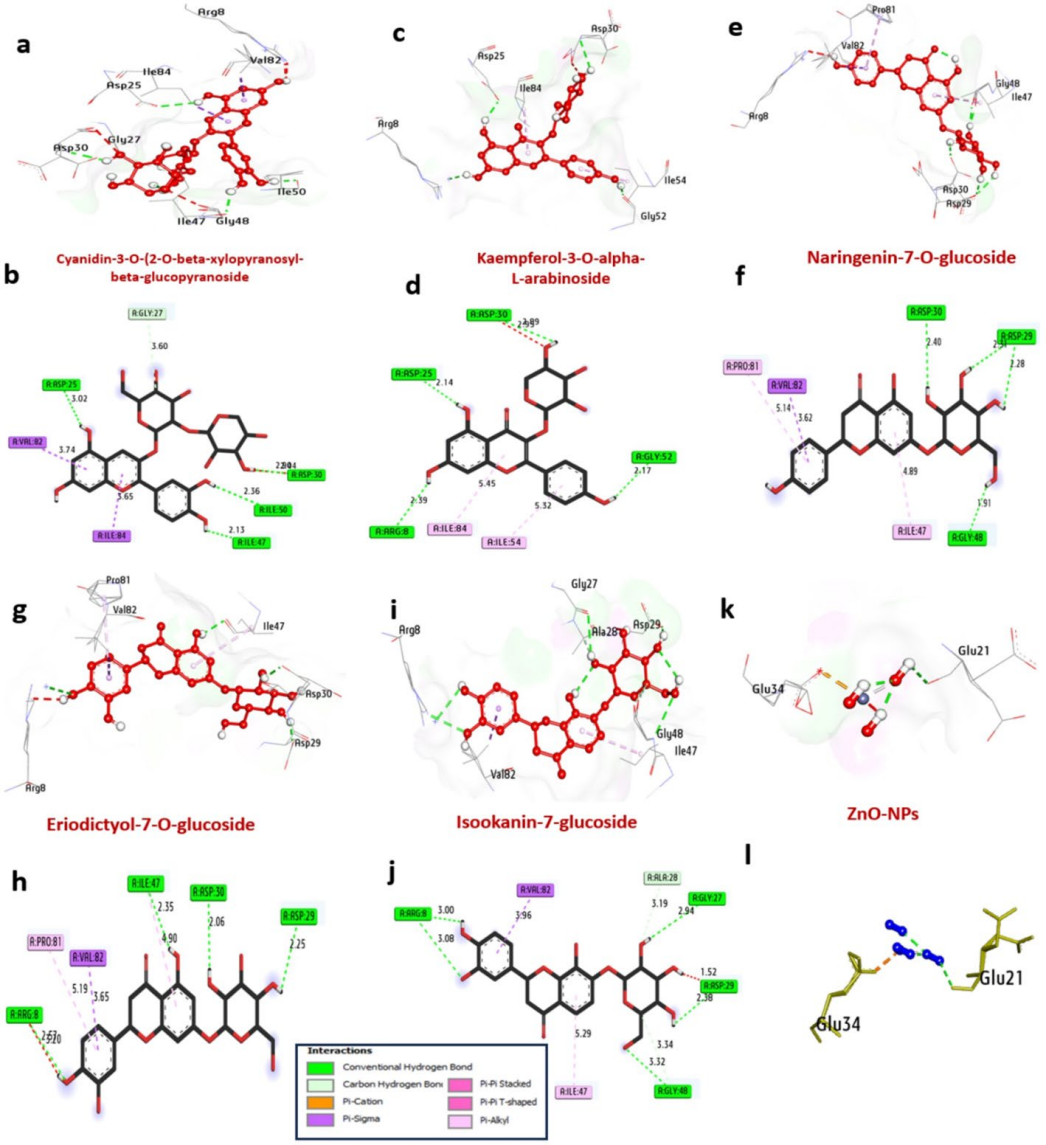
3D representations of compound conformations at the binding pocket of HIV integrase (PDB: ID 5OI2 ):** a**, and** b** Cyanidin-3-O-(2-O-beta-xylopyranosyl-beta-glucopyranoside,** c** and** d** Kaempferol-3-O-alpha-L-arabinoside,** e**, and** f** Naringenin-7-O-glucoside,** g**, and** h** Eriodictyol-7-O-glucoside,** i** and** j** Isookanin-7-glucoside and** k** and** l** ZnO NPs

*HIV-1 Integrase (IN*,* PDB: 5OI2)*: In the catalytic core domain, glycosylated flavonoids demonstrate remarkable potential. Naringenin-7-O-glucoside (− 9.90 kcal/mol) and cyanidin-3-O-xylosylglucoside (− 9.80 kcal/mol) outperformed the control raltegravir (− 9.00 kcal/mol). Their binding was stabilized by multiple hydrogen bonds with the essential catalytic triad residues Asp25, Asp64, and Glu152 (Table 4; Fig. 9). This suggests a mechanism where these compounds could chelate the active site Mg^2+^ ions, blocking viral DNA strand transfer.

**Table 4:** Molecular interactions of ligands with amino acids of HIV integrase (PDB ID: 5OI2):

NO	Protein	Ligand	Hydrophilic interactions		Hydrophobic contacts		No. of H-bonds	No. of Total bonds	affinity kcal mol-1
			Residue (H- bond)	Length	Residue (Bond type)	Length			
1	HIV integrase (PDB: ID 5OI2):		Asp30, (H- Bond) Asp29, (H- Bond) Gly48, (H- Bond) Asp29, (H- Bond)	2.40 2.28 2.31 1.91	Pro81, (alkyl) Ile47, (alkyl) Val82, (Pi-sigma)	5.14 4.89 3.62	**4**	**7**	**-9.90**
		Naringenin-7-O-glucoside							
2		Kaempferol-3-O-alpha-L arabinoside	Asp25, (H- Bond) Gly52, (H- Bond) Arg8, (H- Bond) Asp30, (H- Bond)	2.14 2.17 2.39 2.99	Ile84, (alkyl) Ile54, (alkyl)	5.45 5.32	**4**	**6**	**-9.80**
3		Cyanidin-3-O-(2-O-beta-xylopyranosyl-beta-glucopyranoside	Asp25, (H- Bond) Ile50, (H- Bond) Ile47, (H- Bond) Asp30, (H- Bond)	3.02 2.04 2.36 2.13	Val82, (Pi-sigma) Ile84, (Pi-sigma) Gly27, (Carbon-H bond)	3.74 3.65 3.60	**4**	**7**	**-9.60**

NO	Protein	Ligand	Hydrophilic interactions		Hydrophobic contacts		No. of H-bonds	No. of Total bonds	affinity kcal mol-1
			Residue (H- bond)	Length	Residue (Bond type)	Length			
4	HIV integrase (PDB: ID 5OI2):	Eriodictyol-7-O-glucoside	Asp30, (H- Bond) Asp29, (H- Bond) Ile47, (H- Bond) Arg8, (H- Bond)	2.06 2.25 2.35 2.55	Val82, (Pi-sigma) Pro81, (alkyl) Ile47, (alkyl)	3.65 5.19 4.90	**4**	**7**	**-9.50**
5		Isookanin-7-glucoside	Asp29, (H- Bond) Gly48, (H- Bond) Gly27, (H- Bond) Arg8, (H- Bond) Arg8, (H- Bond)	2.38 3.32 2.94 3.00 3.08	Val82, (Pi-sigma) Ile47, (alkyl Ala28, (Carbon-H bond)	3.96 5.29 3.19	**5**	**8**	**-9.40**
6		ZnO NPs	Glu21, (H- Bond)	2.33	Glu34, (Pi-Cation)	2.50	**1**	**2**	**-9.20**

**Fig. 10:**
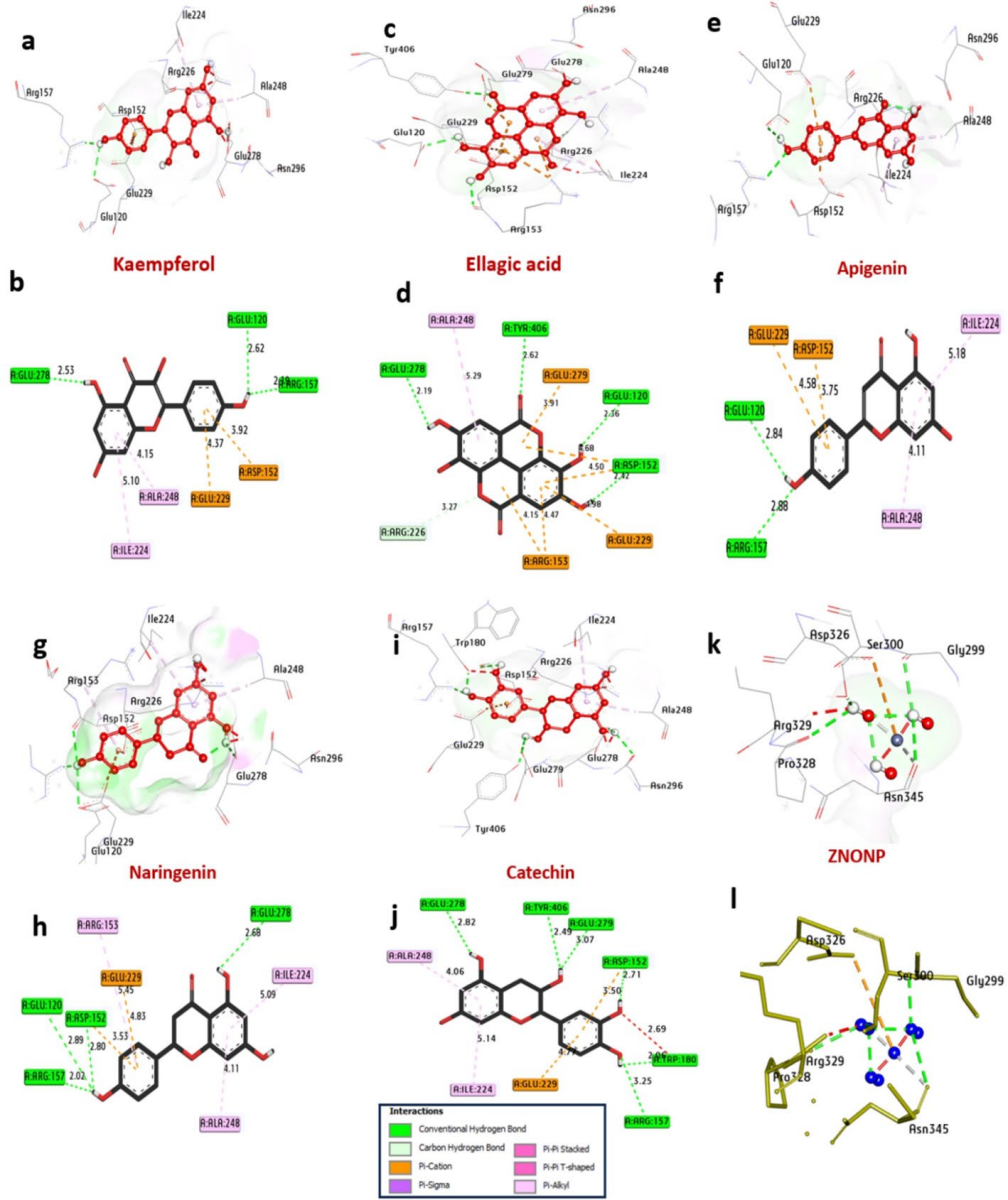
3D representations of compounds at the binding pocket of Neuraminidase of Influenza (PDB: ID 6HCX):** a**, and** b** Kaempferol,** c** and** d** Ellagic acid,** e**, and** f** Apigenin,** g**, and** h** Naringenin,** i** and** j** Catechin and** k** and** l** ZnO NPs

*Influenza A Neuraminidase (NA*,* PDB: 6HCX)*: Docking into the sialic acid binding pocket identified ellagic acid (− 7.70 kcal/mol) and kaempferol (− 7.40 kcal/mol) as strong binders, with affinities comparable to oseltamivir (− 7.20 kcal/mol). These compounds formed critical hydrogen bonds with the conserved catalytic residues Arg118, Glu119, Asp151, and Glu227 (Table 5; Fig. 10), which are vital for the enzyme’s function in viral release.

**Table 5:** Interactions of ligands with neuraminidase of influenza (PDB ID: 6HCX)

No	Protein	Ligand	Hydrophilic interactions		Hydrophobic Contacts		No. of H-bonds	No. of total bonds	affinity kcal mol-1
			Residue (H- bond)	Length	Residue (Bond type)	Length			
1	Neuraminidase of Influenza (PDB: ID 6HCX)	Ellagic acid	Glu278, (H- Bond) Tyr406, (H- Bond) Glu120, (H- Bond) Asp152, (H- Bond)	2.19 2.62 2.36 2.42	Arg226, (Carbon-H bond) Ala248, (Pi-alkyl) Glu279, (Pi-cation) Asp152, (Pi-cation) Asp152, (Pi-cation) Glu229, (Pi-cation) Arg153, (Pi-cation)	3.27 5.29 3.91 4.68 4.50 4.98 4.47	4	11	**-7.70**
2		Kaempferol	Glu120, (H- Bond) Glu278, (H- Bond) Arg157, (H- Bond)	2.62 2.53 2.29	Ala248, (Pi-alkyl) Ile224, (Pi-alkyl) Glu229, (Pi-cation) Asp152, (Pi-cation)	4.15 5.10 2.02 3.92	4	8	**-7.40**
3		Apigenin	Glu120, (H- Bond) Arg157, (H- Bond)	2.84 2.88	Ala248, (Pi-alkyl) Ile224, (Pi-alkyl) Glu229, (Pi-cation) Asp152, (Pi-cation)	4.11 5.18 3.75 4.58	2	6	**-7.30**

No	Protein	Ligand	Hydrophilic interactions		Hydrophobic contacts		No. of H-bonds	No. of total bonds	affinity kcal mol-1
			Residue (H- bond)	Length	Residue (Bond type)	Length			
4	Neuraminidase of Influenza (PDB: ID 6HCX)	Naringenin	Glu278, (H- Bond) Arg157, (H- Bond) Glu120, (H- Bond) Asp152, (H- Bond)	2.68 2.02 2.89 2.80	Ala248, (Pi-alkyl) Ile224, (Pi-alkyl) Arg153, (Pi-alkyl) Glu229, (Pi-cation) Asp152, (Pi-cation)	4.11 5.09 5.45 4.83 3.53	4	9	**-7.30**
5		Catechin	Glu278, (H- Bond) Tyr406, (H- Bond) Glu279, (H- Bond) Trp180, (H- Bond) Asp152, (H- Bond) Arg157, (H- Bond)	2.82 2.49 3.07 2.71 2.06 3.25	Ile224, (Pi-alkyl) Ala248, (Pi-alkyl) Glu229, (Pi-cation) Asp152, (Pi-cation)	5.14 4.06 4.77 3.50	6	10	**-7.10**
6		ZnO NPs	Ser300, (H- Bond) Arg329, (H- Bond) Asn345, (H- Bond)	2.11 3.55 2.11	Asp326, (Pi-cation)	3.05	3	4	**-7.00**

**Fig. 11:**
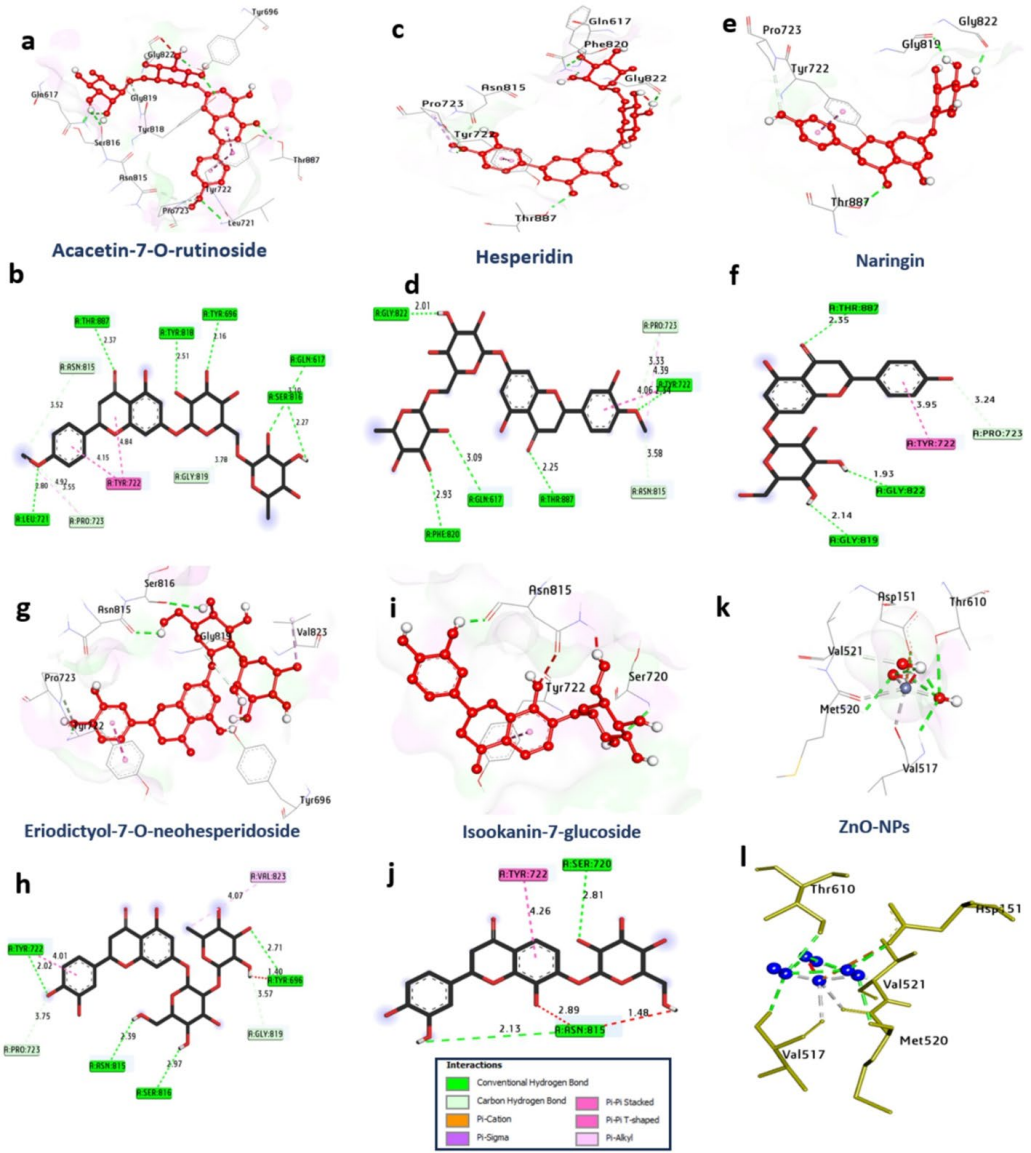
3D representations of compound conformations at the binding pocket of HSV1 polymerase (PDB: ID 7LUF):** a**, and** b** Acacetin-7-O-rutinoside,** c** and** d** Hesperidin,** e**, and** f** Naringin,** g**, and** h** Eriodictyol-7-O-neohesperidoside,** i** and** j** Isookanin-7-glucoside and** k** and** l** ZnO NPs

*HSV-1 DNA Polymerase (PDB: 7LUF)*: Glycosylated flavanones, such as acacetin-7-O-rutinoside (− 8.30 kcal/mol) and hesperidin (− 8.20 kcal/mol), showed promising binding to the polymerase active site, potentially interfering with nucleotide incorporation (Table 6; Fig. 11).

**Table 6:** Molecular interactions with amino acids of HSV1 polymerase (PDB ID: 7LUF)

	Protein	Ligand	Hydrophilic interactions	Hydrophobic contacts			No. of h-bonds	No. of total bonds	Affinity kcal mol-1
			Residue (H- bond)	Length	Residue (Bond type)	Length			
1	HSV1 polymerase (PDB: ID 7LUF)	Acacetin-7-O-rutinoside	Ser816, (H- Bond) Gln617, (H- Bond) Tyr696, (H- Bond) Tyr818, (H- Bond) Thr887, (H- Bond) Leu721, (H- Bond)	2.27 3.10 2.16 2.51 2.37 2.80	Tyr722, (Pi-Pi shaped) Tyr722, (Pi-Pi shaped) Gly819, (CH-bond) Asn815, (CH-bond) Pro723, (CH-bond) Pro723, (Pi-alkyl)	4.84 4.15 3.78 3.52 5.55 4.92	**6**	**12**	**− 8.30**
2		Hesperidin	Gln617, (H- Bond) Tyr722, (H- Bond) Phe820, (H- Bond) Thr887, (H- Bond) Gln617, (H- Bond)	3.09 2.34 2.93 2.25 3.09	Tyr722, (Pi-Pi shaped) Pro723, (CH-bond) Asn815, (CH-bond) Pro723, (Pi-alkyl)	4.06 3.33 3.58 4.39	**5**	**9**	**− 8.20**
3		Naringin	Thr887, (H- Bond) Gly822, (H- Bond) Gly819, (H- Bond)	2.35 1.93 2.14	Tyr722, (Pi-Pi shaped) Pro723, (CH-bond)	3.95 3.24	3	**5**	**− 7.70**

	Protein	Ligand	Hydrophilic Interactions	Hydrophobic contacts			No. of h-bonds	No. of total bonds	Affinity kcal mol-1
			Residue (H- bond)	Length	Residue (Bond type)	Length			
4	HSV1 polymerase (PDB: ID 7LUF)	Eriodictyol-7-O-neohesperidoside	Tyr696, (H- Bond) Ser816, (H- Bond) Asn815, (H- Bond) Tyr722, (H- Bond)	2.71 2.97 2.39 2.02	Tyr722, (Pi-Pi shaped) Pro723, (CH-bond) Gly819, (CH-bond) Val823, (Pi-alkyl)	4.01 3.75 3.57 4.07	**4**	**8**	**− 7.60**
5		Isookanin-7-glucoside	Ser720, (H- Bond) Asn815, (H- Bond)	2.81 2.13	Tyr722, (Pi-Pi shaped)	4.26	**2**	**3**	**− 7.50**
6		ZnO NPs	Thr610, (H- Bond) Val517, (H- Bond) Met520, (H- Bond)	2.33 2.54 4.21	–	–	**3**	**3**	**− 7.50**

To elucidate the molecular mechanisms underlying the observed antiviral activity of the metabolites identified from *C. oliviforme*, in silico molecular docking was performed against four critical viral protein targets. Notably, the docking results suggest that the extract of *C. oliviforme* possesses a significantly higher antiviral potential than *C. cainito*, a superiority attributed to the higher relative concentrations of high-affinity markers—such as chlorogenic acid and quercetin—identified during metabolomic profiling. In summary, the results demonstrated that the major bioactive constituents, specifically apigenin, naringenin-7-O-glucoside, acacetin-7-O-rutinoside, and ellagic acid, exhibited superior binding affinities compared to the standard reference drugs. Detailed interaction analysis revealed that these high negative binding energies indicate spontaneous, robust, and thermodynamically favorable interactions, where ligands stabilize within the catalytic pockets through a synergistic network of hydrogen bonding (with residues like Gln125 and Asp25) and hydrophobic Pi-Pi stacking (with Tyr172 and Tyr722) (Table 7).

**Table 7:** Summary of molecular docking scores and binding affinities

Viral target	Protein target	PDB ID	Top-scoring ligand	Binding energy (kcal/mol)	Reference drug
Herpes simplex (HSV)	Thymidine kinase	2KI5	Apigenin	− 10.80	Acyclovir
HIV-1	Integrase	5OI2	Naringenin-7-O-glucoside	− 9.90	Raltegravir
Adenovirus	DNA polymerase	7LUF	Acacetin-7-O-rutinoside	− 8.30	Cidofovir
Influenza (H5N1)	Neuraminidase	6HCX	Ellagic acid	− 7.70	Oseltamivir

#### In silico pharmacokinetic and toxicity (ADMET) profiling

**Fig. 12:**
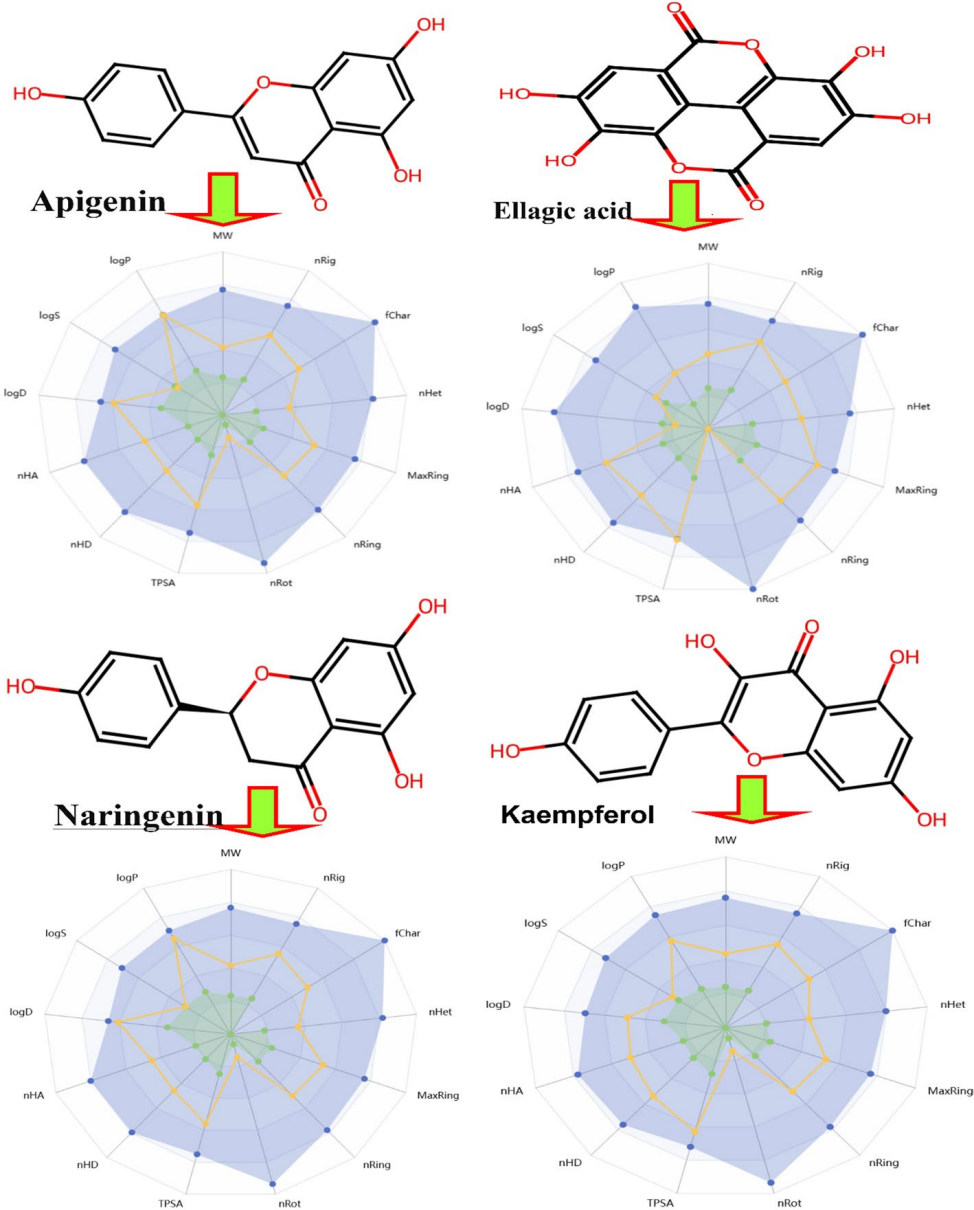
Oral bioavailability graph for compounds

To assess the drug-likeness of the lead docked compounds (apigenin, ellagic acid, naringin, kaempferol), in silico ADMET predictions were performed (Table 8; Fig. 12). Apigenin and kaempferol exhibited excellent drug-like properties, adhering to Lipinski’s Rule of Five (MW < 500, LogP < 5, H-bond donors ≤ 5, acceptors ≤ 10), with moderate lipophilicity (LogP ~ 2) and high predicted gastrointestinal absorption. Ellagic acid had favorable properties but lower lipophilicity (LogP 1.28), which might limit membrane permeability. Naringin, due to its high molecular weight (580.92 g/mol) and glycosylation, violated Lipinski’s rules and had poor predicted blood-brain barrier penetration, but showed high solubility.

**Table 8:** Prediction of the pharmacokinetics and physicochemical properties of the compounds

Id	ID	Apigenin	Ellagic acid	Naringin	Kaempferol	Id	ID	Apigenin	Ellagic acid	Naringin	Kaempferol
Physicochemical Properties	MW	270.05	302.01	272.07	286.05	Metabolism	CYP1A2-inh	1.000	0.904	0.586	0.997
	Vol	265.19	265.70	267.82	273.98		CYP1A2-sub	0.470	0.009	0.678	0.598
	Dense	1.02	1.14	1.02	1.04		CYP2C19-inh	0.112	0.000	0.526	0.132
	nHA	5.00	8.00	5.00	6.00		CYP2C19-sub	0.000	0.000	0.001	0.001
	nHD	3.00	4.00	3.00	4.00		CYP2C9-inh	0.002	0.028	0.948	0.799
	TPSA	90.90	141.34	86.99	111.13		CYP2C9-sub	0.673	0.564	0.158	0.519
	nRot	1.00	0.00	1.00	1.00		CYP2D6-inh	0.957	0.000	0.016	0.005
	nRing	3.00	4.00	3.00	3.00		CYP2D6-sub	1.000	0.035	0.856	0.995
	MaxRing	10.00	14.00	10.00	10.00		CYP3A4-inh	1.000	0.004	0.999	0.975
	nHet	5.00	8.00	5.00	6.00		CYP3A4-sub	0.000	0.000	0.000	0.002
	Fear	0.00	0.00	0.00	0.00	Excretion	CL (Clearance)	5.939	14.743	6.894	5.694
	nRig	18.00	21.00	18.00	18.00		T12	1.203	1.648	1.312	1.388
	Flex	0.06	0.00	0.06	0.06	Toxicity	hERG Blockers	0.100	0.028	0.107	0.069
	nStereo	0.00	0.00	1.00	0.00		H-HT	0.545	0.210	0.520	0.478
Solubility	LogS	− 4.216	− 3.369	− 4.021	− 3.648		DILI	0.743	0.984	0.220	0.703
	LogD	2.567	0.752	2.678	1.931		AMES Toxicity	0.618	0.670	0.703	0.546
	LogP	2.981	0.951	2.596	1.965		Rat OralToxicity	0.520	0.509	0.500	0.488
	ESOL Log S	− 3.94	− 2.94	− 3.49	− 3.31		FDAMDD	0.882	0.632	0.747	0.805
	Ali Log S	− 4.59	− 3.66	− 3.99	− 3.86		Skin Sensitization	0.645	0.895	0.746	0.621
	Silicon-IT class	Soluble	Soluble	Soluble	Soluble		Carcinogenicity	0.793	0.666	0.591	0.716
drug-likeness	Lipinski Rule	Accepted	Accepted	Accepted	Accepted		Eye Corrosion	0.371	0.032	0.039	0.575
	Pfizer rule	Accepted	Accepted	Accepted	Accepted		Eye Irritation	0.998	0.967	0.997	0.998
	Golden triangle	Accepted	Accepted	Accepted	Accepted		Respiratory Toxicity	0.777	0.277	0.266	0.713

Id	ID	Apigenin	Ellagic acid	Naringin	Kaempferol	Id	ID	Apigenin	Ellagic acid	Naringin	Kaempferol
Absorption	Pgp-inh	0.004	0.000	0.956	0.142	Toxicophoric Rules	Non-Genotoxic Carcinogenicity	1	2	1	
	Pgp-sub	0.312	0.041	0.186	0.163		LD50_oral	3.692	3.839	3.712	3.653
	HIA	0.002	0.500	0.000	0.015		Neurotoxicity-DI	0.061	0.003	0.644	0.039
	F (20%)	0.565	0.953	0.002	0.084		Ototoxicity	0.068	0.372	0.251	0.075
	F (30%)	0.837	0.967	0.925	0.716		Hematotoxicity	0.043	0.154	0.075	0.045
	Caco-2	− 5.129	− 5.167	− 4.987	− 5.969		Nephrotoxicity-DI	0.021	0.039	0.328	0.019
	MDCK	− 4.759	− 4.743	− 4.762	− 4.909		Genotoxicity	0.987	0.953	0.978	0.977
Distribution	BBB	0.013	0.001	0.000	0.001	Medicinal Chemistry	RPMI-8226	0.045	0.031	0.099	0.040
	PPB%	96.535	68.544	94.987	97.881		QED	0.63	0.22	0.74	0.55
	VDss	− 0.463	− 0.353	− 0.011	− 0.812		Synth	2.25	2.98	2.83	2.38
	Fu %	3.858	24.246	7.285	1.360		Fsp3	0.00	0.00	0.13	0.00

All compounds were predicted to have low risks of AMES mutagenicity, hepatotoxicity, and skin sensitization, supporting their safety profiles as lead molecules (Table 9).

**Table 9:** Prediction of toxicity risks and oral toxicity prediction results of the compounds

No	Ligand	Toxicity risks				Physicochemical properties					
		Mutagenic	Tumorigenic	Irritant	Reproductive	CLogP	Solubility	Molecular Weight	TPSA	Drug likeness	Drug score
1	Apigenin	(+)	(−)	(−)	(−)	2.34	− 2.86	270.0	86.90	1.21	0.47
2	Ellagic acid	(-)	(−)	(−)	(−)	1.28	− 3.29	302.0	133.6	− 1.60	0.51
3	Naringin	(-)	(−)	(−)	(−)	2.16	− 2.64	272.0	86.99	1.90	0.84
4	Kaempferol	(+)	(−)	(−)	(−)	1.84	− 2.79	286.0	107.2	0.90	0.46

#### Molecular dynamics simulation (MDS) for binding stability

**Fig. 13:**
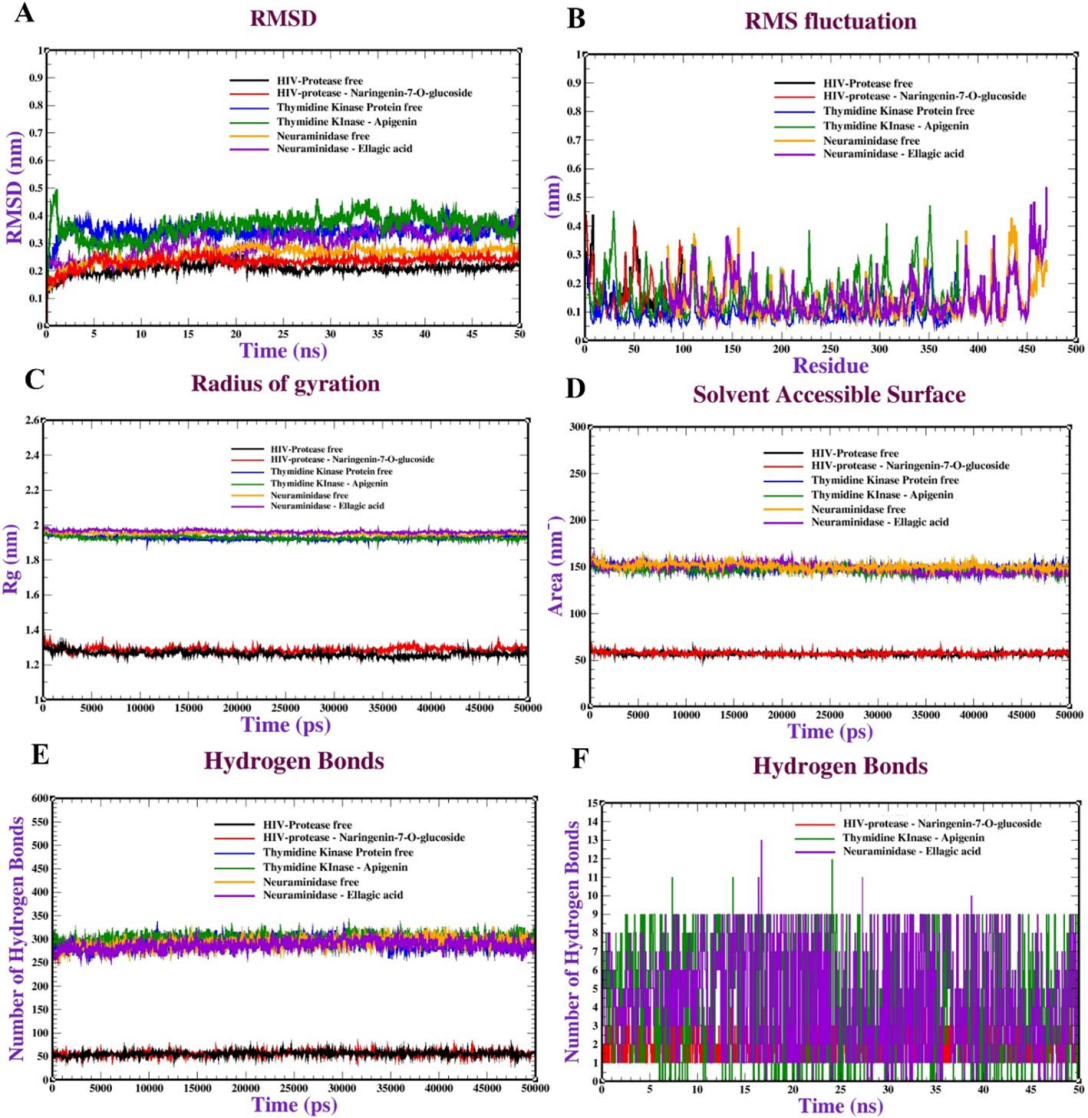
Molecular dynamics of HIV protease with naringenin-7-O-glucoside, thymidine kinase with apigenin, and neuraminidase with ellagic acid:** A** RMSD,** B** RMSF,** C** Radius of gyration (Rg),** D** SASA,** E** Intramolecular hydrogen bonds and** F** Intermolecular hydrogen bonds

To validate the docking poses and assess the long-term stability of the key protein-ligand complexes, MD simulations were conducted for a total duration of 50 ns using GROMACS 2018 (Fig. 13). In summary, the analysis focused on the top-ranking complexes: HIV-1 Integrase with Naringenin-7-O-glucoside, Thymidine Kinase with Apigenin, and Neuraminidase with Ellagic Acid. The Root Mean Square Deviation (RMSD) of the protein backbones and ligand atoms confirmed robust binding, with the systems reaching structural equilibrium and plateauing after approximately the first 10 ns. Notably, the RMSD values remained consistently low (ranging from 0.10 to 0.35 nm), indicating stable complex formation with minimal conformational drift throughout the remainder of the 50 ns trajectory.

Furthermore, the Root Mean Square Fluctuation (RMSF) analysis revealed that ligand binding significantly reduced flexibility in the active site loops, effectively “locking” the enzymes into a conformation less favorable for viral catalysis. Residue fluctuations remained remarkably low, typically between 0.15 and 0.4 nm. To provide a concise overview of structural integrity, the Radius of Gyration (Rg) remained constant (1.25–1.95 nm across targets), confirming that the proteins maintained their compact, folded, and globular states without major unfolding events. Altogether, the persistence of these interactions was further supported by a consistently high number of hydrogen bonds and reduced Solvent Accessible Surface Area (SASA). these MDS results provide high-confidence validation that the identified phytochemicals from *C. oliviforme* form stable, long-lasting complexes with viral targets, strongly supporting their potential as genuine antiviral inhibitors.

### Computational validation

To ensure the accuracy and reliability of the findings, the computational protocols were subjected to a rigorous multi-step validation process. Initially, target preparation was meticulously performed using PyMOL, where protein structures were pre-processed to remove all water molecules and native ligands, ensuring the docking grid was focused exclusively on known catalytic sites to eliminate non-specific binding artifacts. Subsequently, the docking protocol underwent formal redocking validation by repositioning co-crystallized ligands back into their respective active sites; the resulting predicted poses showed high structural overlap with experimental crystallographic data, thereby confirming the precision of the AutoDock Vina parameters. Altogether, these *in* silico models demonstrated a clear biological correlation, as the high negative binding energies directly mirrored the potent antiviral activity observed in the plaque reduction assays. Notably, this was evidenced by the high Selectivity Indices (SI), such as the SI of 8.02 for biogenic ZnO NPs against H5N1 providing robust empirical support for the theoretical binding mechanisms identified in this study.

## Discussion

The present study provides a holistic, comparative analysis of two *Chrysophyllum* species, systematically linking their inherent phytochemical wealth to their functional capacity for nanomaterial synthesis and antiviral bioactivity. The results paint a compelling picture of *C. oliviforme* as a phytochemically superior and functionally more potent species compared to *C. cainito*.

### Ecological and biosynthetic implications of distinct metabolomic profiles

The LC-HRMS/MS data reveal not just quantitative, but qualitative differences in the secondary metabolomes of *C. oliviforme* and *C. cainito*. The dominance of flavonoids, particularly in *C. oliviforme*, aligns with the ecological roles of these compounds as UV photoprotectants, antioxidants, and antimicrobial agents. The significantly higher accumulation (15–57%) of specific metabolites in *C. oliviforme* quercetin, chlorogenic acid, rosmarinic acid, resveratrol, esculetin—suggests a more activated or differently regulated phenylpropanoid pathway. This could be an adaptive response to heightened biotic (pathogen/herbivore pressure) and abiotic (intense solar radiation, soil composition) stressors in its native tropical habitat compared to that of *C. cainito* [29, 30]. The shift towards flavonol production in *C. oliviforme* versus flavanones in *C. cainito* points to a differential expression or activity of key enzymes like flavonol synthase (FLS) versus flavanone 3-hydroxylase (F3H). This interspecific metabolic channeling has direct implications for bioactivity, as flavonols like quercetin are often more potent antioxidants than their flavanone precursors [31, 32].

The identification of 86 metabolites, including many previously unreported for these species, significantly expands the known chemical space of the genus *Chrysophyllum*. The presence of specialized metabolites like resveratrol (a stilbene renowned for its cardioprotective and anti-aging properties) and cyanidin-based anthocyanins in *C. oliviforme* is particularly noteworthy [33, 34]. It elevates this species from a mere source of generic phenolics to a potential reservoir of high-value, nutraceutical-grade compounds. This chemoprofile directly correlates with proposed health benefits: the flavonol and phenolic acid abundance suggests strong antioxidant and anti-inflammatory potential [35, 36], while resveratrol and specific flavan-3-ols (catechin, epicatechin) are linked to cardiovascular protection [37, 38].

### Phytochemistry as a blueprint for nanomaterial engineering

The “green synthesis” of nanoparticles is not a generic process; it is a biologically templated reduction and stabilization event dictated by the specific chemistry of the extract. The superior physicochemical properties of *C. oliviforme* derived ZnO NPs smaller size, lower PDI, higher negative zeta potential, and enhanced crystallinity, are a direct consequence of its superior phytochemical profile.

The mechanism can be deconstructed as follows: The reduction of Zn^2+^ to Zn^0^ is facilitated by electron-donating groups like phenolic -OH. *C. oliviforme*’s higher concentration of reductants like quercetin and chlorogenic acid likely provides a more rapid and uniform nucleation burst, leading to a larger number of smaller seed particles [39, 40]. Subsequently, the stabilization and capping phase is governed by molecules that can coordinate with the nanoparticle surface. Here, the diverse array of polyphenols, flavonoids, and possibly proteins in *C. oliviforme* extract acts as a multi-dentate capping layer. The strong negative zeta potential (− 45.8 mV) is a testament to the ionization of surface-bound phenolic acids (e.g., chlorogenic acid dissociates to carboxylate anions), creating a powerful electrostatic repulsion barrier [41]. In contrast, the less rich and potentially different compositional blend in *C. cainito* extract results in slower reduction, less effective capping, and consequently, larger, more polydisperse, and less stable NPs (PDI 0.41, Zeta − 37.1 mV).

The enhanced crystallinity of *C. oliviforme* NPs, as evidenced by sharper XRD peaks, is another critical finding. It suggests that the phytochemical milieu in this extract does not merely cap the particles but may also moderate the growth kinetics, allowing for more orderly atomic addition and fewer crystal defects. This has profound implications for the functional properties of the NPs, as crystallinity directly influences optoelectronic properties, photocatalytic activity, and even biological interactions [42, 43]. Thus, selecting *C. oliviforme* over *C. cainito* for synthesis is not just a matter of degree but of engineering a fundamentally higher-quality nanomaterial.

### Deciphering the antiviral mode of action: synergy, Trade-offs, and mechanistic insights

The antiviral results present a nuanced picture of potency versus selectivity. The consistent outperformance of *C. oliviforme*, both as an extract and as an NP source, can be traced back to its chemoprofile. Its extract’s excellent activity against influenza H5N1 (SI = 8.02) may be attributed to the synergy of its rich flavonoid content (e.g., kaempferol, which docks strongly to neuraminidase) with other constituents that may inhibit viral entry or replication at other stages.

The transition from crude extract to ZnO NP introduces a paradigm shift in the mode of action. The enhanced potency of the NPs is multifactorial due to the intrinsic Activity of ZnO: ZnO ions and surfaces can generate reactive oxygen species (ROS), damage viral envelopes, and inhibit viral replication cycles [44, 45]. Synergistic Phytochemical Delivery: The non-calcined, green-synthesized NPs retain a corona of bioactive phytochemicals from the extract. This creates a synergistic entity where the NP acts as a carrier, potentially concentrating and delivering phytochemicals like apigenin or ellagic acid directly to the site of viral infection, while the phytochemicals enhance NP stability and biocompatibility [46–48]. Multi-target Potential: While the crude extract’s compounds may hit individual viral targets (e.g., TK, polymerase), the ZnO NP-phytochemical conjugate could simultaneously engage in physical virucidal damage (membrane disruption), ROS-based attack, and specific enzymatic inhibition, creating a multi-pronged antiviral strategy [49–51].

However, this enhanced power comes at a cost: increased cytotoxicity. The higher surface area and reactivity of the NPs, along with the potential for Zn^2+^ ion leaching, can induce oxidative stress and damage host cell membranes [52, 53]. This is reflected in the lower SI values of the NPs compared to the safer, but less potent, crude extracts. This trade-off defines a critical avenue for future work: the functionalization or coating of these green-synthesized NPs to mitigate cytotoxicity while retaining antiviral synergy, for instance, by embedding them in a biodegradable polymer or further engineering the phytochemical capping layer.

### Computational validation and future drug development pathways

The in-silico studies move beyond correlation to propose concrete mechanisms. The docking results are not merely a computational exercise; they provide a rational filter to identify which of the 86 detected metabolites are most likely to contribute to the observed antiviral effects. The strong binding of apigenin to HSV TK, ellagic acid to influenza NA, and glycosylated flavonoids to HIV IN offers testable hypotheses for isolating specific active principles.

The ADMET predictions further refine this list. Apigenin and kaempferol stand out not only for their strong docking scores but also for their excellent predicted drug-likeness and safety profiles. They represent prime candidates for isolation, in vitro enzymatic assays, and further preclinical development as standalone antiviral leads. The MDS results are the final, crucial piece of computational validation. The stability of the complexes over 50 ns of simulated biological motion confirms that the docking poses are not artifacts but represent thermodynamically favorable, persistent interactions. This greatly increases confidence that these compounds would perform as inhibitors in a physiological setting [54, 55].

The study of the ZnO NP model in docking, while simplified, is a pioneering step. It acknowledges that the NP entity itself may have specific molecular interactions, potentially blocking active sites through steric hindrance or interacting with key residues via its surface chemistry.

### Broader implications, limitations, and future directions

This research positions *C. oliviforme* as a promising, sustainable resource for developing standardized phytopharmaceuticals and for the eco-friendly production of functional nanomaterials with built-in bioactivity. The established link between metabolome and nanomaterial properties argues for the “fingerprinting” of plant extracts as a prerequisite for predictable green synthesis.

However, several limitations remain and point to clear directions for future research: although molecular docking indicates potential enzyme inhibition, the precise stage of viral interference whether during entry, replication, assembly, or release requires experimental confirmation using time-of-addition assays and visualization approaches such as TEM to elucidate virus–nanoparticle interactions; moreover, the reported synergy between ZnO nanoparticles and phytochemicals warrants quantitative clarification to determine whether the effect is merely additive or truly synergistic, which could be addressed through extract fractionation and evaluation of individual fractions in nanoparticle synthesis and bioactivity assays to identify the key capping agents; in addition, the encouraging in vitro and in silico findings must be validated in suitable animal models to assess pharmacokinetics, antiviral efficacy, and systemic safety; finally, further optimization of nanoparticle design, including surface coating or functionalization strategies, is necessary to minimize cytotoxicity while preserving antiviral potency.

## Conclusion

This integrated study demonstrates that *C. oliviforme* possesses a metabolomic profile distinct from and superior to that of *C. cainito*, characterized by a greater abundance of bioactive flavonoids, phenolic acids, and specialized metabolites. This phytochemical supremacy directly translates into the biosynthesis of higher-quality ZnO nanoparticles with superior size, stability, and crystallinity. Both species exhibit broad-spectrum antiviral activity, with *C. oliviforme* consistently showing greater potency and selectivity, most notably against influenza H5N1. The enhanced activity of the green-synthesized ZnO NPs, albeit with a cytotoxicity trade-off, highlights a promising synergistic strategy. Computational analyses successfully identified specific phytoconstituents (e.g., apigenin, ellagic acid) as putative viral enzyme inhibitors and validated their stable binding, providing a molecular foundation for the observed bioactivity. These findings champion *C. oliviforme* as a valuable species for nutraceutical exploration and as an effective, sustainable bio-agent for engineering functional nanomaterials with inherent antiviral properties, and suggest potential for multi-target nano-therapeutics, though further in vivo studies are required to address the inherent cytotoxicity of zinc-based nanomaterials.

## Supplementary Information

Below is the link to the electronic supplementary material.

Supplementary Material 1.

## Data Availability

All data generated or analyzed during this study are included in this published article and its supplementary information files. Raw LC-MS data and antiviral assay datasets are available from the corresponding author upon reasonable request.
