# Supplementary material for: Superiority of Chrysophyllum oliviforme in the green synthesis of highly stable ZnO nanoparticles: metabolomic profiling, quadruple antiviral screening, and comparative MD simulations
Source: Discov Nano. 2026 Mar 9;21(1):61. doi: 10.1186/s11671-026-04459-z (PMC12972423; doi:10.1186/s11671-026-04459-z)
Supplement: Supplementary file 1 — Supplementary Material 1. [file 11671_2026_4459_MOESM1_ESM.docx]

***Superiority of Chrysophyllum oliviforme in the Green Synthesis of Highly Stable ZnO Nanoparticles: Metabolomic Profiling, Quadruple Antiviral Screening, and Comparative MD Simulations****.*

**Mina Michael Melk^1^*, and Ahmed F. El-Sayed^2, 3^**

^1^ Pharmacognosy Department, Faculty of Pharmacy, Ahram Canadian University, Giza, Egypt; [mina.michael@acu.edu.eg](mailto:mina.michael@acu.edu.eg) (M.M.M.)

^2^Microbial Genetics Department, Biotechnology Research Institute, National Research Centre, Giza, Egypt; af.rizk@nrc.sci.e.g. (A.F.S.)

^3^Egypt Center for Research and Regenerative Medicine (ECRRM), Cairo, Egypt; ahmedfikry.nrc@gmail.com (A.F.S.)

*Correspondence: [mina.michael@acu.edu.eg](mailto:mina.michael@acu.edu.eg) (M.M.M.)


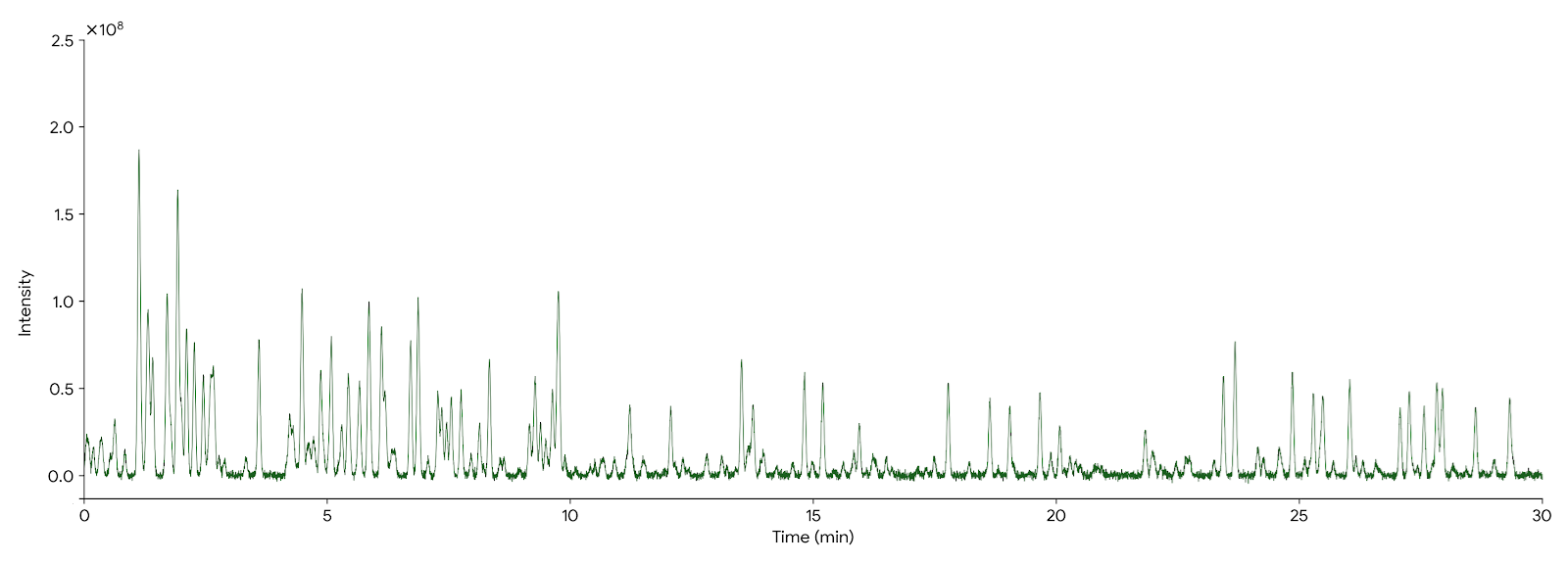


Fig. S1: LC-MSMS chromatogram of *C. oliviform*e


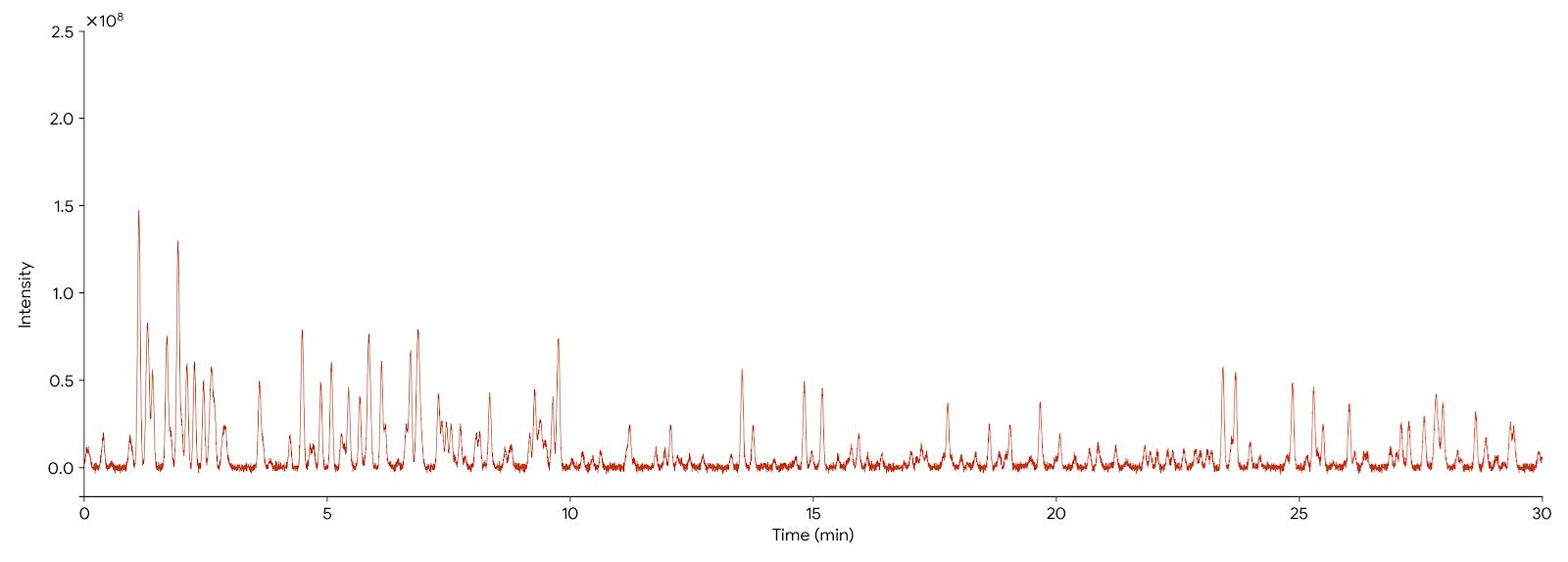


Fig. S2: LC-MSMS chromatogram of *C. cainito*

**Table S1. Phenolic compounds identified by LC‒MS-MS from the extracts of the leaves of *C. oliviforme and C. cainito*.**

| Number | T(min) | [M-H]⁻ (Da) | Error(ppm) | Compound | Top 3 MS/MS Fragments (m/z) | Relative Intensity *C. oliviforme* | Relative Intensity *C. cainito* | Ref |
| --- | --- | --- | --- | --- | --- | --- | --- | --- |
|  | 1.115 | 317.0302 | 0.9 | Myricetin | 151.0034, 179.0340, 271.0251 | 4.76 | 3.03 | [1] |
|  | 1.125 | 353.0878 | 0.6 | Chlorogenic Acid | 191.0556, 173.0454, 135.0441 | 11.43 | 9.7 | [1] |
|  | 1.14 | 163.0399 | 0.3 | o-Coumaric Acid | 119.0492, 93.0340, 163.0400 | 2.86 | 1.82 | [1] |
|  | 1.158 | 109.0289 | 1.1 | Catechol | 81.0335, 108.0210, 109.0289 | 1.9 | 1.21 | [1] |
|  | 1.29 | 287.0556 | 0.7 | Eriodictyol | 125.0233, 151.0034, 287.0559 | 3.81 | 4.24 | [2, 3, 4] |
|  | 1.3 | 153.0187 | 0.2 | 3,4-Dihydroxybenzoic Acid | 109.0284, 108.0209, 153.0188 | 2.86 | 1.82 | [1] |
|  | 1.323 | 577.1551 | 1.1 | Rhoifolin | 289.0708, 245.0809, 577.1553 | 5.71 | 4.85 | [1] |
|  | 1.406 | 447.0926 | 0.7 | Quercitrin | 300.0267, 301.0342, 151.0034 | 6.67 | 5.45 | [1] |
|  | 1.701 | 285.0399 | 0.4 | Luteolin | 133.0284, 151.0034, 285.0401 | 7.62 | 6.06 | [1] |
|  | 1.721 | 163.0399 | 0.3 | p-Coumaric Acid | 119.0492, 93.0340, 163.0400 | 3.81 | 2.42 | [1] |
|  | 1.78 | 175.0395 | 0.6 | 7-Hydroxy-4-methylcoumarin | 117.0337, 89.0389, 175.0399 | 2.86 | 1.82 | [1] |
|  | 1.916 | 301.0348 | 0.5 | Hesperetin | 164.0109, 137.0239, 286.0478 | 4.76 | 3.64 | [1] |
|  | 1.916 | 609.1451 | 1 | Luteolin diglucoside | 285.0399, 447.0928, 609.1453 | 5.71 | 4.85 | [2, 3, 4] |
|  | 1.941 | 463.0876 | 0.6 | Isoquercitrin | 300.0267, 271.0249, 151.0034 | 6.67 | 5.45 | [1] |
|  | 2.006 | 401.1083 | 1 | Sinapic acid glucoside | 223.0608, 169.0139, 385.1152 | 3.81 | 2.42 | [2, 5] |
|  | 2.103 | 609.1818 | 1.2 | Eriodictyol glycoside | 287.0556, 331.0602, 443.1750 | 4.76 | 3.64 | [2, 3, 4] |
|  | 2.115 | 449.1082 | 1 | Okanin glucoside | 287.0556, 169.0139, 271.0249 | 3.81 | 2.42 | [2, 5] |
|  | 2.263 | 289.0716 | 0.8 | (+)-Catechin | 125.0233, 203.0809, 289.0719 | 7.62 | 6.06 | [1] |
|  | 2.455 | 477.0983 | 0.7 | Isorhamnetin glucoside | 314.0420, 151.0034, 477.0985 | 5.71 | 4.85 | [1] |
|  | 2.607 | 507.1089 | 1 | Syringetin galactoside | 344.0528, 169.0139, 507.1091 | 4.76 | 3.64 | [1] |
|  | 2.642 | 299.0552 | 0.8 | Kaempferol derivative | 151.0034, 285.0399, 299.0554 | 3.81 | 2.42 | [2, 3, 4] |
|  | 2.678 | 137.0237 | 0.4 | p-Hydroxybenzoic acid | 93.0336, 109.0284, 137.0239 | 2.86 | 1.82 | [4] |
|  | 3.59 | 480.1359 | 1.3 | Glucoerucin | 178.9921, 259.0119, 480.1362 | 1.9 | 1.21 | [5] |
|  | 3.608 | 461.0719 | 0.8 | Kaempferol glucuronide | 285.0399, 113.0238, 461.0722 | 4.76 | 3.64 | [1] |
|  | 4.232 | 153.0187 | 0.2 | 2,5-Dihydroxybenzoic acid | 109.0284, 125.0233, 153.0188 | 2.86 | 1.82 | [5] |
|  | 4.474 | 289.0716 | 0.8 | (-)-Epicatechin | 125.0233, 203.0809, 289.0719 | 7.62 | 6.06 | [1] |
|  | 4.501 | 415.1028 | 0.9 | Daidzein C-glucoside | 253.0498, 415.1030, 133.0284 | 3.81 | 2.42 | [1] |
|  | 4.71 | 183.0029 | 0.5 | 3,4-Dihydroxymandelate | 123.0081, 124.0157, 183.0031 | 1.9 | 1.21 | [5] |
|  | 4.867 | 359.0765 | 0.7 | Rosmarinic acid | 161.0239, 179.0340, 359.0768 | 5.71 | 4.85 | [1] |
|  | 5.084 | 455.0989 | 1 | FMN | 377.0661, 455.0991, 96.9609 | 1.9 | 1.21 | [5] |
|  | 5.087 | 477.0721 | 0.5 | Quercetin glucuronide | 301.0348, 151.0034, 477.0723 | 5.71 | 4.85 | [1] |
|  | 5.288 | 177.019 | 0.4 | Esculetin | 133.0284, 105.0340, 177.0192 | 2.86 | 1.82 | [1] |
|  | 5.428 | 623.1612 | 1.1 | Isorhamnetin rutinoside | 315.0507, 300.0267, 623.1615 | 4.76 | 3.64 | [1] |
|  | 5.466 | 393.2009 | 1.2 | Lysophosphatidic acid | 279.2329, 393.2012, 152.9954 | 1.9 | 1.21 | [5] |
|  | 5.663 | 595.1298 | 1 | Quercetin arabinoglucoside | 300.0267, 301.0342, 595.1301 | 4.76 | 3.64 | [1] |
|  | 5.853 | 625.1405 | 1.1 | Quercetin diglucoside | 300.0267, 463.0878, 625.1408 | 5.71 | 4.85 | [1] |
|  | 5.865 | 431.0979 | 0.6 | Apigenin C-glucoside | 269.0455, 283.0249, 431.0982 | 3.81 | 2.42 | [1] |
|  | 5.893 | 197.045 | 0.5 | Vanillylmandelic acid | 137.0239, 150.0109, 197.0452 | 1.9 | 1.21 | [4] |
|  | 6.117 | 577.1296 | 1 | Kaempferol coumaroylglucoside | 285.0399, 255.0293, 577.1299 | 4.76 | 3.64 | [1] |
|  | 6.119 | 611.1604 | 1.2 | Delphinidin rutinoside | 303.0501, 465.1033, 611.1607 | 3.81 | 2.42 | [2, 4] |
|  | 6.189 | 581.1498 | 1.1 | Cyanidin xylosylglucoside | 287.0556, 449.1084, 581.1501 | 3.81 | 2.42 | [1] |
|  | 6.714 | 289.0716 | 0.8 | Catechin | 125.0233, 203.0809, 289.0719 | 7.62 | 6.06 | [1] |
|  | 6.863 | 433.0772 | 0.6 | Quercetin xyloside | 300.0267, 433.0775, 151.0034 | 4.76 | 3.64 | [1] |
|  | 6.881 | 447.0926 | 0.7 | Kaempferol glucoside | 285.0399, 447.0929, 151.0034 | 5.71 | 4.85 | [1] |
|  | 7.286 | 447.0926 | 0.7 | Quercetin rhamnoside | 300.0267, 447.0929, 151.0034 | 4.76 | 3.64 | [1] |
|  | 7.362 | 417.0822 | 0.6 | Kaempferol arabinoside | 285.0399, 417.0825, 151.0034 | 3.81 | 2.42 | [1] |
|  | 7.457 | 389.1239 | 1 | Stilbene glucoside | 227.0709, 389.1242, 152.9863 | 2.86 | 1.82 | [2, 5] |
|  | 7.547 | 433.1134 | 0.9 | Naringenin glucoside | 271.0609, 433.1137, 151.0034 | 3.81 | 2.42 | [1] |
|  | 7.747 | 463.1184 | 1 | Peonidin glucoside | 301.0348, 463.1187, 151.0034 | 3.81 | 2.42 | [1] |
|  | 8.142 | 191.0556 | 0.5 | Quinic acid | 93.0336, 147.0449, 191.0559 | 2.86 | 1.82 | [1] |
|  | 8.336 | 591.1662 | 1.1 | Acacetin rutinoside | 283.0609, 591.1665, 151.0034 | 3.81 | 2.42 | [1] |
|  | 8.338 | 435.1292 | 0.9 | Phlorizin | 273.0758, 435.1295, 125.0233 | 2.86 | 1.82 | [1] |
|  | 9.163 | 449.1082 | 1 | Isookanin glucoside | 287.0556, 449.1085, 151.0034 | 2.86 | 1.82 | [1] |
|  | 9.268 | 445.077 | 0.8 | Baicalein glucuronide | 269.0455, 445.0773, 113.0238 | 3.81 | 2.42 | [1] |
|  | 9.286 | 193.0349 | 0.4 | Galacturonic acid | 85.0288, 193.0352, 93.0336 | 1.9 | 1.21 | [3] |
|  | 9.384 | 403.1395 | 1 | Stilbene glucoside | 241.0859, 403.1398, 179.0340 | 2.86 | 1.82 | [2, 5] |
|  | 9.502 | 407.2899 | 1.4 | Cholic acid | 343.2378, 407.2902, 108.9476 | 1.9 | 1.21 | [5] |
|  | 9.637 | 433.0772 | 0.6 | Quercetin arabinoside | 300.0267, 433.0775, 151.0034 | 4.76 | 3.64 | [1] |
|  | 9.736 | 227.0712 | 0.7 | Resveratrol | 185.0601, 227.0715, 143.0493 | 3.81 | 2.42 | [1] |
|  | 9.761 | 301.0348 | 0.5 | Quercetin | 151.0034, 179.0340, 301.0351 | 7.62 | 6.06 | [1] |
|  | 11.228 | 315.0505 | 0.8 | Isorhamnetin | 300.0267, 315.0508, 151.0034 | 3.81 | 2.42 | [1] |
|  | 12.069 | 431.0979 | 0.6 | Kaempferol rhamnoside | 285.0399, 431.0982, 151.0034 | 3.81 | 2.42 | [1] |
|  | 13.531 | 285.0399 | 0.4 | Kaempferol | 151.0034, 285.0402, 133.0284 | 6.67 | 5.45 | [1] |
|  | 13.771 | 315.0505 | 0.8 | Tamarixetin | 300.0267, 315.0508, 151.0034 | 3.81 | 2.42 | [3, 5] |
|  | 14.815 | 269.0455 | 0.5 | Apigenin | 117.0337, 151.0034, 269.0458 | 5.71 | 4.85 | [1] |
|  | 15.191 | 283.0607 | 0.6 | Acacetin | 117.0337, 151.0034, 283.0610 | 4.76 | 3.64 | [1] |
|  | 15.947 | 339.0716 | 0.8 | Esculin | 177.0190, 339.0719, 119.0492 | 2.86 | 1.82 | [1] |
|  | 17.773 | 271.0607 | 0.6 | Naringenin | 151.0034, 177.0189, 271.0610 | 4.76 | 3.64 | [1] |
|  | 18.632 | 179.0344 | 0.3 | Caffeic acid | 135.0441, 179.0347, 134.9512 | 3.81 | 2.42 | [1] |
|  | 19.046 | 449.1082 | 1 | Eriodictyol glucoside | 287.0556, 449.1085, 151.0034 | 3.81 | 2.42 | [2, 3, 5] |
|  | 19.664 | 463.0876 | 0.6 | Myricitrin | 300.0267, 463.0879, 151.0034 | 4.76 | 3.64 | [1] |
|  | 20.065 | 167.0344 | 0.3 | DOPAC | 122.0366, 167.0347, 123.0441 | 2.86 | 1.82 | [5] |
|  | 21.825 | 147.0446 | 0.5 | Cinnamic acid | 103.0542, 147.0449, 102.9748 | 1.9 | 1.21 | [1] |
|  | 23.431 | 609.1451 | 1 | Rutin | 300.0267, 609.1454, 151.0034 | 5.71 | 4.85 | [1] |
|  | 23.691 | 285.0399 | 0.4 | Kaempferol | 151.0034, 285.0402, 133.0284 | 6.67 | 5.45 | [1] |
|  | 24.855 | 609.1818 | 1.2 | Hesperidin | 301.0348, 609.1821, 151.0034 | 5.71 | 4.85 | [1] |
|  | 25.291 | 579.1711 | 1.1 | Naringin | 271.0609, 579.1714, 151.0034 | 4.76 | 3.64 | [1] |
|  | 25.489 | 169.0137 | 0.3 | Gallic acid | 125.0233, 169.0140, 123.0081 | 3.81 | 2.42 | [1] |
|  | 26.035 | 300.9983 | 0.8 | Ellagic acid | 229.0009, 300.9986, 151.0034 | 4.76 | 3.64 | [1] |
|  | 27.086 | 193.0501 | 0.7 | Ferulic acid | 134.0366, 193.0504, 117.0337 | 3.81 | 2.42 | [1] |
|  | 27.264 | 223.0607 | 0.8 | Sinapic acid | 208.0379, 223.0610, 131.0208 | 3.81 | 2.42 | [5] |
|  | 27.565 | 163.0399 | 0.3 | p-Coumaric acid | 119.0492, 163.0400, 115.0055 | 3.81 | 2.42 | [1] |
|  | 27.82 | 305.0661 | 0.8 | Gallocatechin | 125.0233, 305.0664, 151.0034 | 4.76 | 3.64 | [3, 5] |
|  | 27.95 | 305.0661 | 0.8 | Epigallocatechin | 125.0233, 305.0664, 151.0034 | 4.76 | 3.64 | [3, 5] |
|  | 28.635 | 153.0187 | 0.2 | Protocatechuic acid | 109.0284, 153.0188, 107.0176 | 3.81 | 2.42 | [1] |
|  | 29.334 | 197.045 | 0.5 | Syringic acid | 182.0218, 197.0453, 121.0029 | 3.81 | 2.42 | [1] |


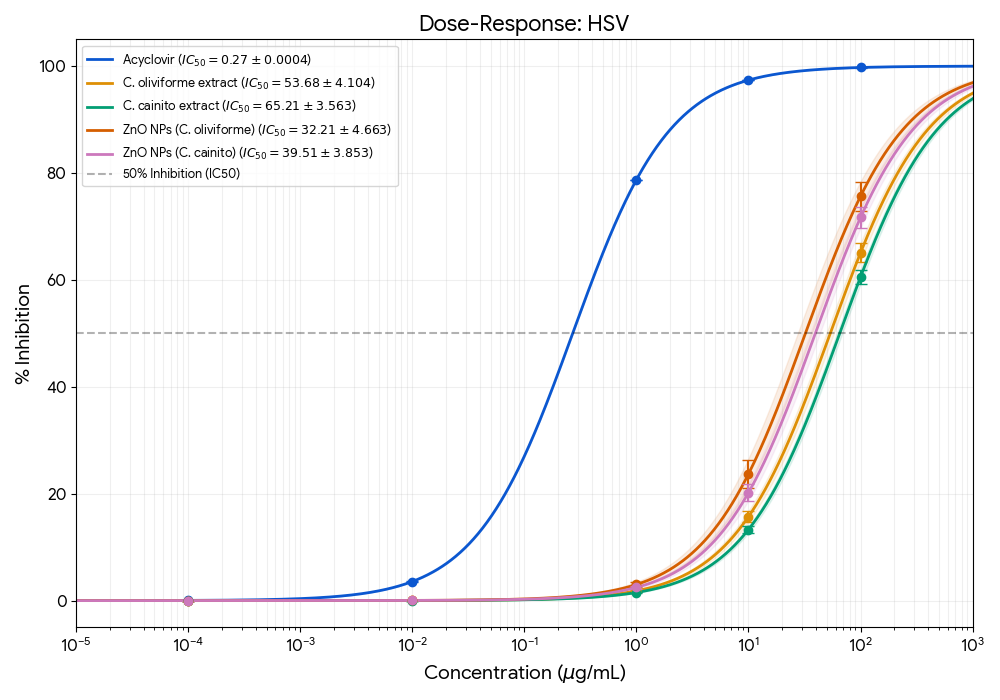


Fig. S3: Antiviral dose response curve of HSV


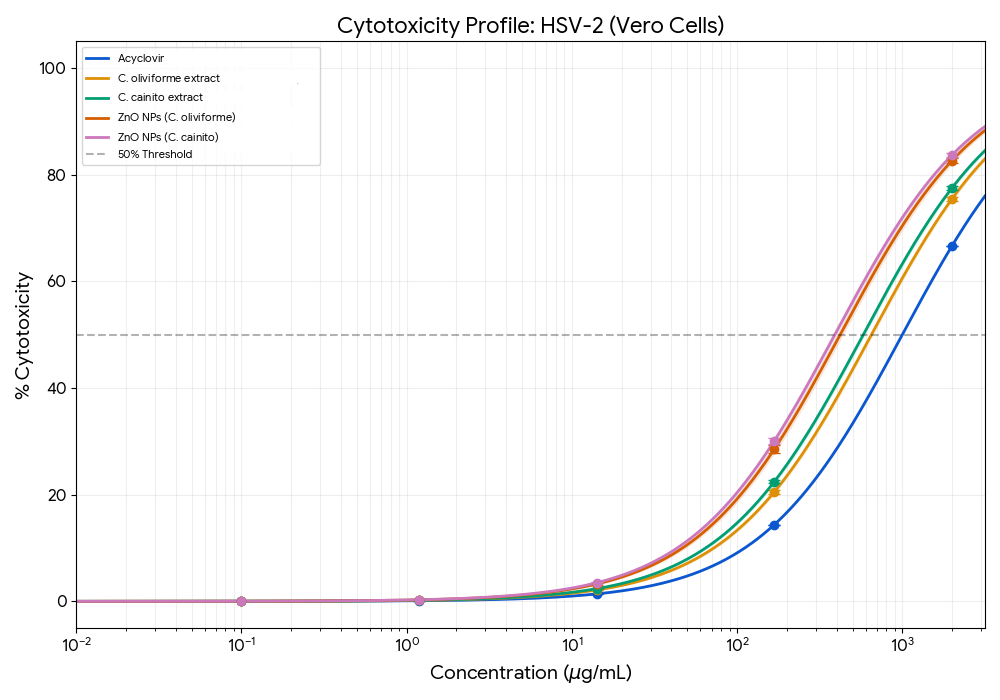


Fig. S4: Cytotoxic dose response curve of HSV


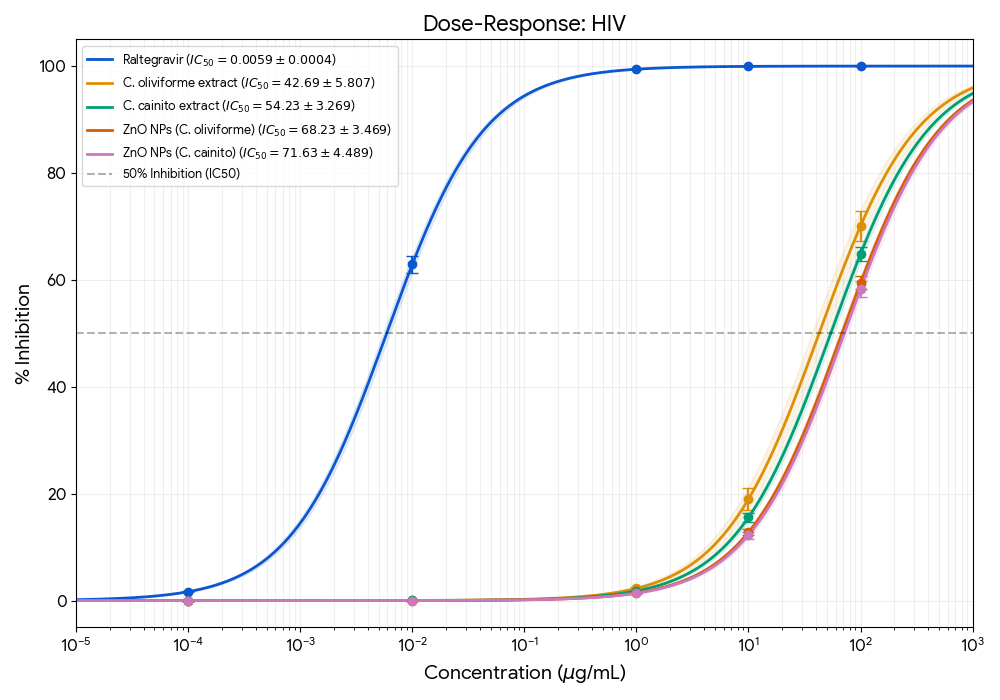


Fig. S5: Antiviral dose response curve of HIV


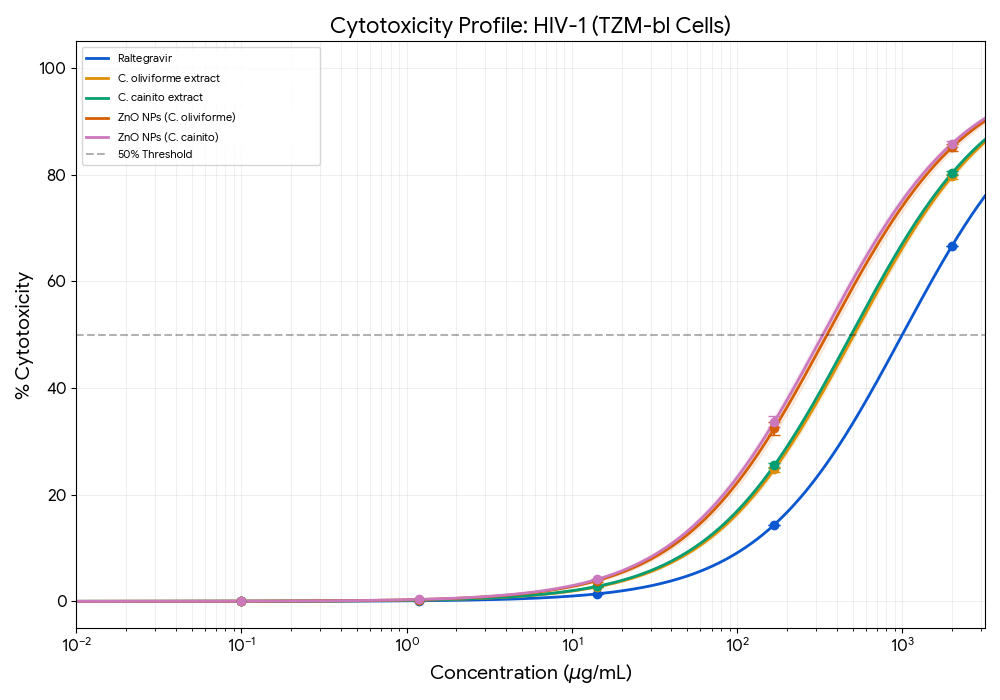


Fig. S6: Cytotoxic dose response curve of HIV


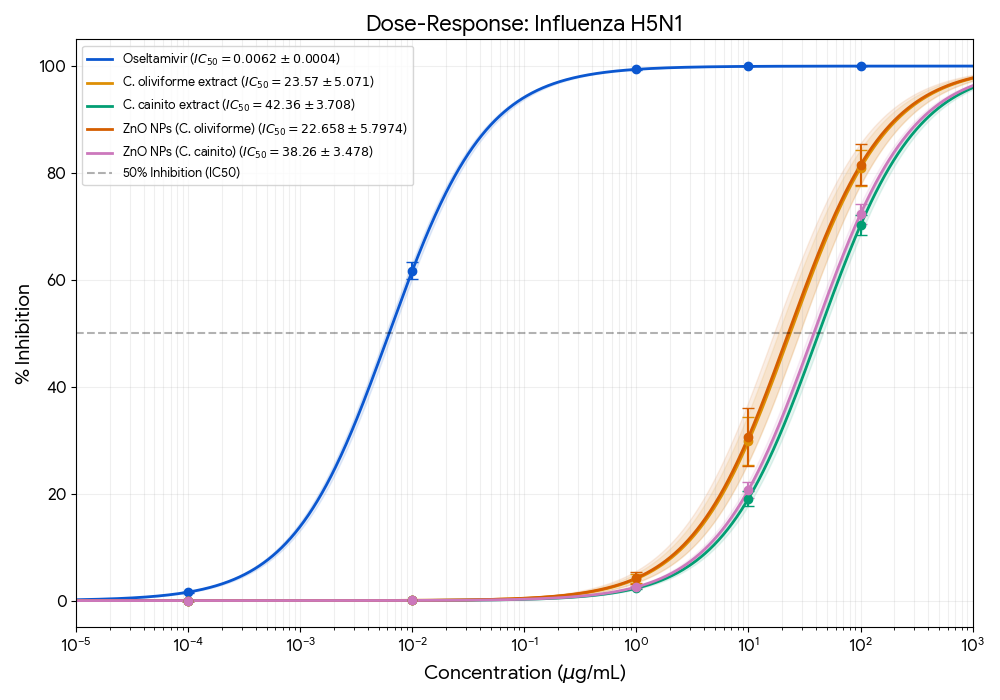


Fig. S7: Antiviral dose response curve of H5N1


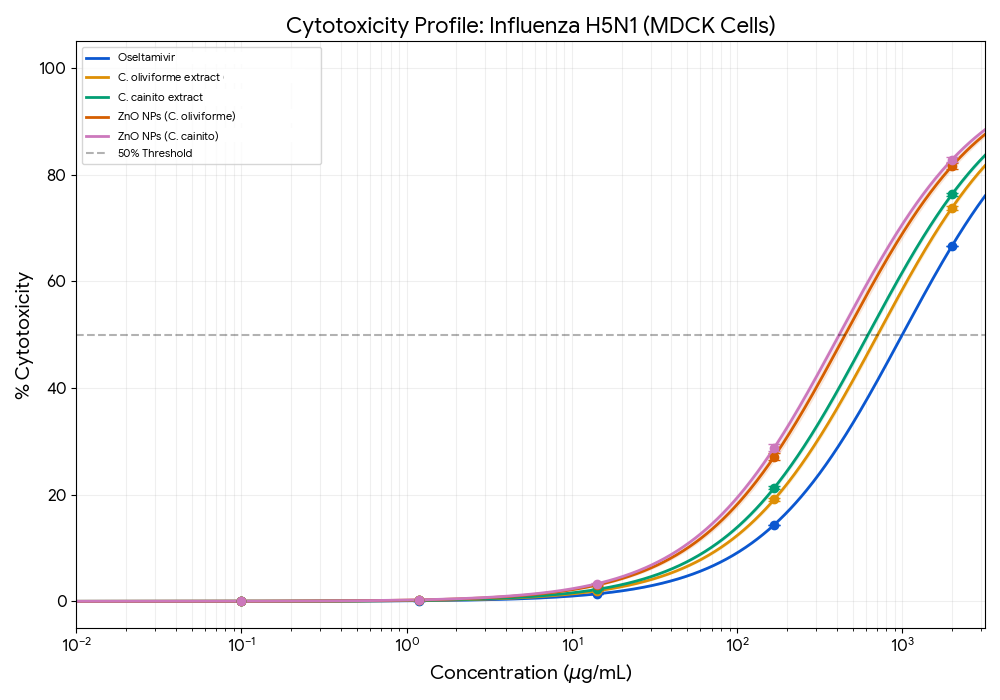


Fig. S8: Cytotoxic dose response curve of H5N1


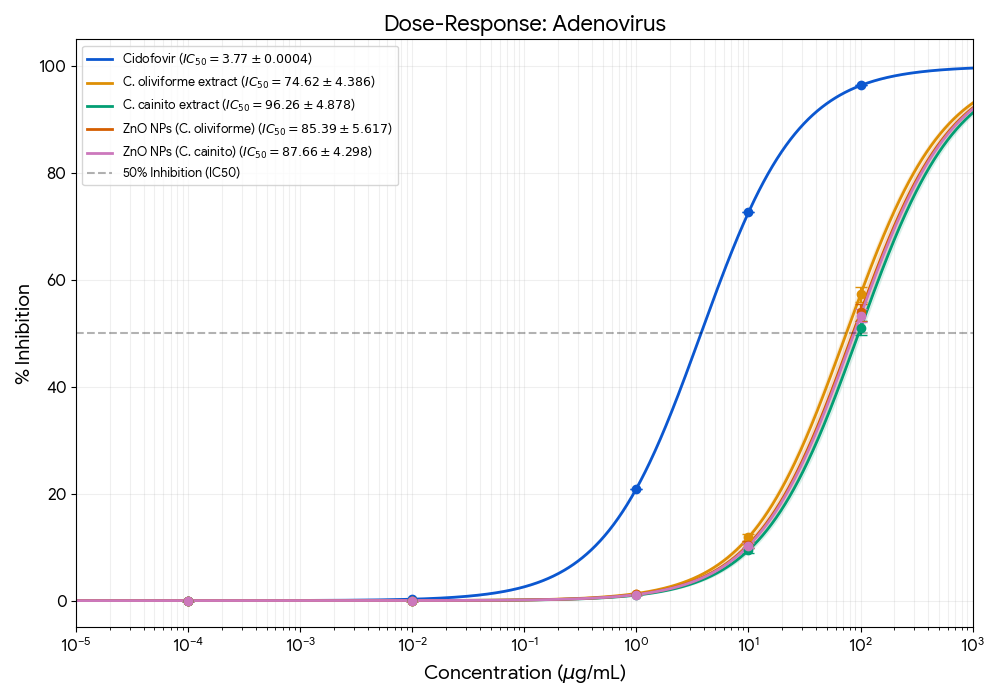


Fig. S9: Antiviral dose response curve of Adenovirus-40


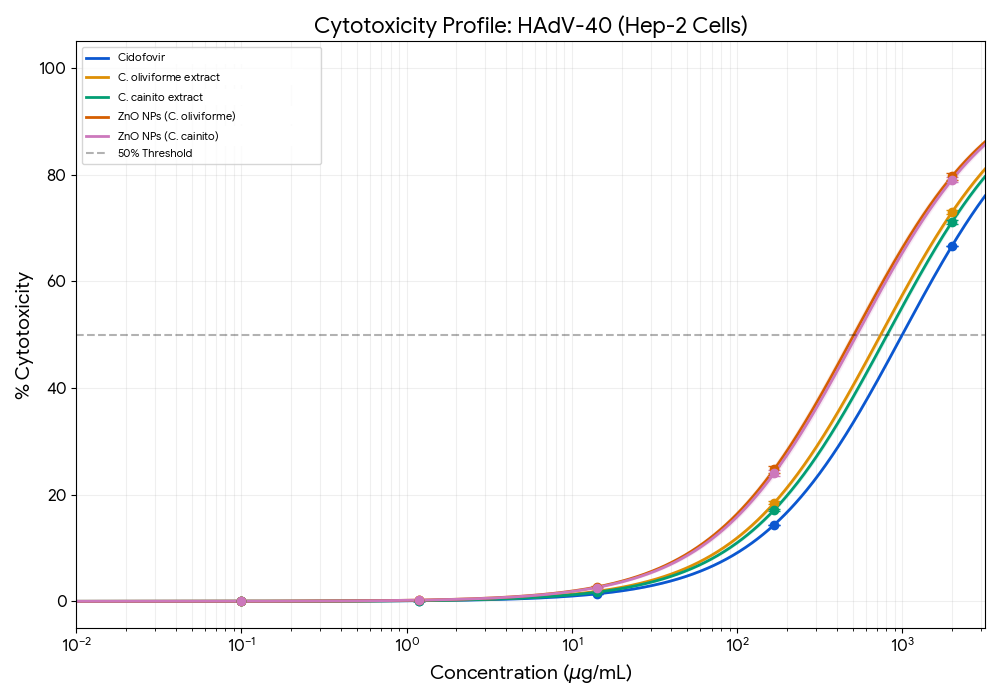


Fig. S10: Cytotoxic dose response curve of Adenovirus-40

SupplementaryReferences

1. Melk MM, El-Sayed AF. Phytochemical profiling, antiviral activities, molecular docking, and dynamic simulations of selected *Ruellia* species extracts. *Sci Rep*. 2024;14(1):15381.
2. Du T, Wang Y, Xie H, Liang D, Gao S. Fragmentation patterns of phenolic C-glycosides in mass spectrometry analysis. *Molecules*. 2024;29(13):2953. <https://doi.org/10.3390/molecules29132953>
3. Wang Y, Gu M, Mao J, Liu J, Fan S, Zhang H, et al. Phytochemical study: Fragmentation patterns of flavonoid-C-glycosides in the enriched flavonoids from corn silk using high-efficiency ultrahigh-performance liquid chromatography combined with quadrupole time-of-flight mass spectrometry. *Separation Science Plus*. 2024;7(2):2300156.
4. Kachlicki P, Piasecka A, Stobiecki M, Marczak Ł. Structural characterization of flavonoid glycoconjugates and their derivatives with mass spectrometric techniques. *Molecules*. 2016;21(11):1494. <https://doi.org/10.3390/molecules21111494>
5. Huang B, Chen F, Zhang X, Hu Y, Zhang Y, Chen L, Wen P. A fragmentation study of disaccharide flavonoid C-glycosides using triple quadrupole mass spectrometry and its application for identification of flavonoid C-glycosides in *Odontosoria chinensis*. *Rapid Commun Mass Spectrom*. 2025;39(2):e9936.
